# Transforming microbial pigment into therapeutic revelation: extraction and characterization of pyocyanin from *Pseudomonas aeruginosa* and its therapeutic potential as an antibacterial and anticancer agent

**DOI:** 10.1186/s12934-024-02438-6

**Published:** 2024-06-13

**Authors:** Moustafa A. Marey, Rania Abozahra, Nefertiti A. El-Nikhely, Miranda F. Kamal, Sarah M. Abdelhamid, Mohammed A. El-Kholy

**Affiliations:** 1 Department of Microbiology and Biotechnology, Division of Clinical and Biological Sciences, College of Pharmacy, Arab Academy for Science, Technology and Maritime Transport (AASTMT), Abu Kir Campus, P.O. Box 1029, Alexandria, Egypt; 2 Microbiology and Immunology Department, Faculty of Pharmacy, Damanhour University, Damanhour, Egypt; 3 Department of Biotechnology, Institute of Graduate Studies and Research, Alexandria University, Alexandria, Egypt; 4 Department of Pharmaceutical Analytical Chemistry, Faculty of Pharmacy, Damanhour University, Beheira, Egypt

**Keywords:** *Pseudomonas aeruginosa*, Pyocyanin, *phzM*, *phzS*, Phenazine derivatives, Bioactive compounds, Therapeutic potential, Antibacterial, Anticancer

## Abstract

**Background:**

The objectives of the current study were to extract pyocyanin from *Pseudomonas aeruginosa* clinical isolates, characterize its chemical nature, and assess its biological activity against different bacteria and cancer cells. Due to its diverse bioactive properties, pyocyanin, being one of the virulence factors of *P. aeruginosa*, holds a promising, safe, and available therapeutic potential.

**Methods:**

30 clinical *P. aeruginosa* isolates were collected from different sources of infections and identified by routine methods, the VITEK 2 compact system, and 16 S rRNA. The phenazine-modifying genes (*phzM*, *phzS*) were identified using polymerase chain reaction (PCR). Pyocyanin chemical characterization included UV-Vis spectrophotometry, Fourier Transform Infra-Red spectroscopy (FTIR), Gas Chromatography-Mass Spectrometry (GC-MS), and Liquid Chromatography-Mass Spectrometry (LC-MS). The biological activity of pyocyanin was explored by determining the MIC values against different clinical bacterial strains and assessing its anticancer activity against A549, MDA-MB-231, and Caco-2 cancer cell lines using cytotoxicity, wound healing and colony forming assays.

**Results:**

All identified isolates harboured at least one of the *phzM* or *phzS* genes. The co-presence of both genes was demonstrated in 13 isolates. The UV-VIS absorbance peaks were maxima at 215, 265, 385, and 520 nm. FTIR could identify the characteristic pyocyanin functional groups, whereas both GC-MS and LC-MS elucidated the chemical formula C_11_H_18_N_2_O_2_, with a molecular weight 210. The quadri-technical analytical approaches confirmed the chemical nature of the extracted pyocyanin. The extract showed broad-spectrum antibacterial activity, with the greatest activity against *Bacillus*, *Staphylococcus*, and *Streptococcus* species (MICs 31.25–125 µg/mL), followed by *E. coli* isolates (MICs 250–1000 µg/mL). Regarding the anticancer activity, the pyocyanin extract showed IC_50_ values against A549, MDA-MB-231, and Caco-2 cancer cell lines of 130, 105, and 187.9 µg/mL, respectively. Furthermore, pyocyanin has markedly suppressed colony formation and migratory abilities in these cells.

**Conclusions:**

The extracted pyocyanin has demonstrated to be a potentially effective candidate against various bacterial infections and cancers. Hence, the current findings could contribute to producing this natural compound easily through an affordable method. Nonetheless, future studies are required to investigate pyocyanin’s effects in vivo and analyse the results of combining it with other traditional antibiotics or anticancer drugs.

**Supplementary Information:**

The online version contains supplementary material available at 10.1186/s12934-024-02438-6.

## Background

The extreme high number of cancer patients and spread of antimicrobial resistance (AMR) are two of the most serious challenges that threaten global health [1]. Despite significant advancements in oncology, cancer continues to be one of the main causes of death globally [2]. According to the world health organisation (WHO), cancer is the most leading cause of mortality before the age of 70 in many countries [3]. It is one of the most dreaded health issues on Earth, where around 19.3 million new cases and 10 million deaths from cancer were recorded globally in 2020. Furthermore, the incidence rate is expected to increase by 47% to reach 28.4 million cases worldwide by 2040. The most commonly diagnosed cancers are female breast cancer, lung cancer, and colorectal cancer. Lung cancer is still on the top of the list of cancer-related deaths, with colorectal cancer coming in second, and female breast cancer in fifth place [4]. The WHO listed antimicrobial resistance as one of the top ten public health issues impacting humankind in 2019. It was estimated that drug-resistant illnesses kill at least 700,000 people worldwide each year, whereas rates are expected to rise to reach 10 million by 2050 [5]. Antibiotic resistance has been highlighted by the World Economic Forum as a global problem that cannot be managed by any nation or institution [6]. Consequently, there is an ongoing need to develop a novel class of more affordable and safer natural compounds with high selectivity and specificity against cancer as well as microbial infections [7].

Nowadays, microorganisms have gained attention for being biological agents that can combat many health-related problems owing to producing arsenals of bioactive compounds as secondary metabolites such as toxins, alkaloids, antibiotics, and pigments [8, 9]. Moreover, natural and eco-friendly bio-pigments have drawn a lot of interests due to their high safety profile, increasing consumer acceptance, and their ability to reduce health-related problems [10]. Among all natural sources, bacterial pigments are an appealing target and more desirable than other plant or animal sources. This is attributed to their rapid development, sustainable availability, and simplicity of controlling microbial cell factories for high production yields. In addition, they have showed diverse biological activities including antimicrobial, anticancer, and anticoagulant effects [11]. Phenazines are a crucial class of these pigments, which are produced by some microorganisms; the most common is *Pseudomonas aeruginosa* [12].

*P. aeruginosa* is a Gram-negative pathogen that causes serious nosocomial infections in the urinary tract, circulatory system, neurological system, respiratory system, and other body systems [13–16]. *P. aeruginosa* produces several virulence factors known as phenazines. Pyocyanin is an example of phenazines, which has been thoroughly studied to date and is emerging as a virulence factor of interest given the antimicrobial resistance and chronic nature of *Pseudomonas* infections [17]. Nonetheless, *P. aeruginosa* is one of the most beneficial microbes in biotechnology applicability because it secretes a wide range of pigments, including redox-active phenazine compounds. In addition to being virulence factors, phenazines are secondary metabolites with beneficial biological activities and applications. Phenazines include pyocyanin (blue-green), which is the most significant substance in this category, pyoverdine (yellow and fluorescent), pyorubrin (red-brown), and pyomelanin (light-brown) [11, 18].

**Fig. 1:**
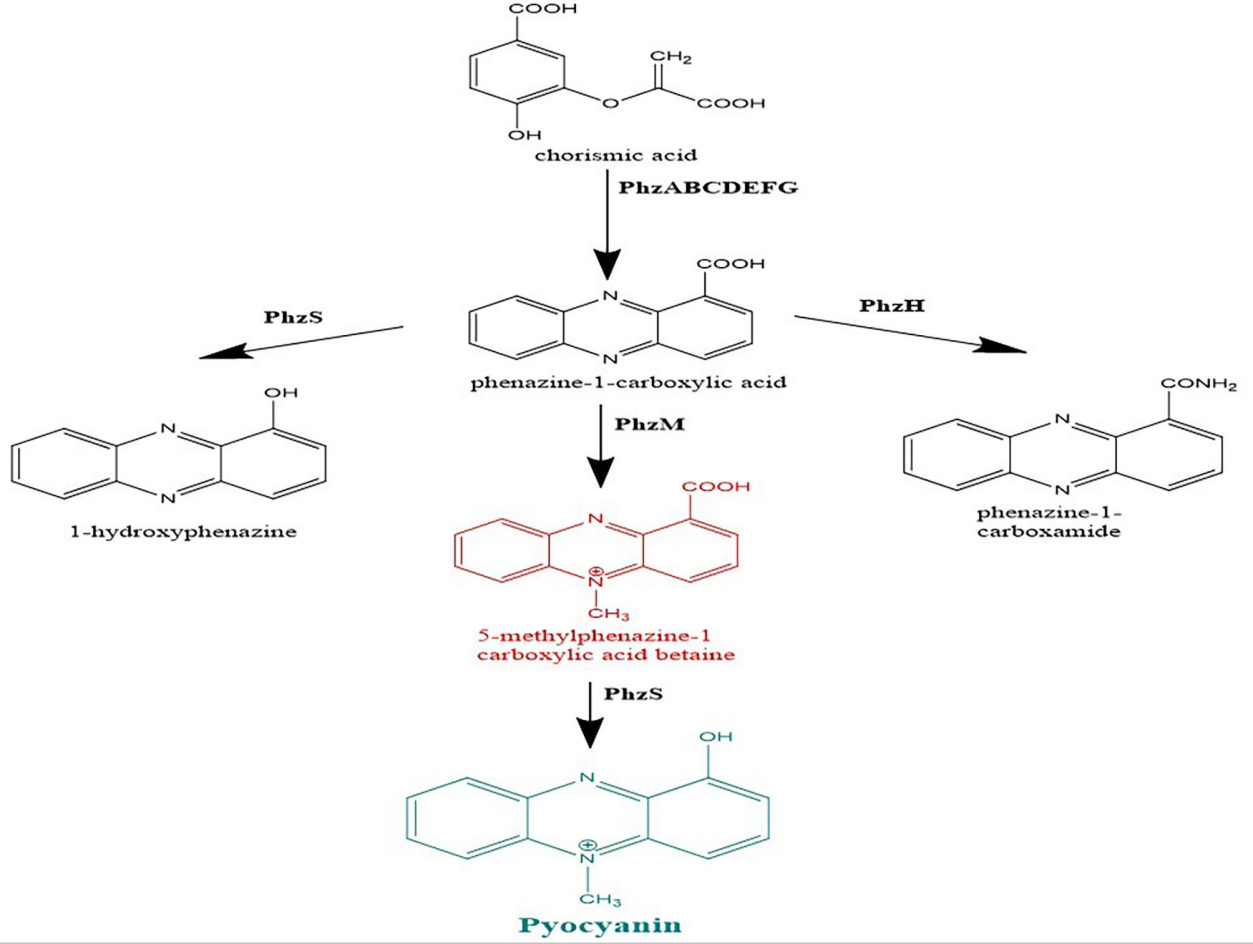
Biosynthesis pathway of pyocyanin from chorismic acid in *Pseudomonas aeruginosa*

As shown in Fig. 1, the biosynthesis of pyocyanin begins with chorismic acid (CA), which is regarded as pyocyanin’s precursor. Seven genes, encoded by two operons (*phzA1B1C1D1E1F1G1* and *phzA2B2C2D2E2F2G2*), regulate the conversion of CA to phenazine-1-carboxylic acid (PCA). Then, the synthesis of pyocyanin from PCA is controlled by two phenazine - modifying genes, *phzM, and phzS*. PCA is transformed into 5-methylphenazine-1-carboxylic acid betaine (MPCBA), using a phenazine - specific methyltransferase (PhzM). The final step is the decarboxylation of MPCBA by flavin-dependent monooxygenase (PhzS), which yields pyocyanin. Therefore, *phzM* and *phzS* are the two crucial genes responsible for pyocyanin biosynthesis [12, 19].

Pyocyanin is regarded as a virulence factor as well as a quorum-sensing signalling molecule involved in several key bioprocesses. Having hydrophilic and hydrophobic characteristics, pyocyanin interacts with the membrane of sensitive prokaryotic and eukaryotic cells, causing oxidative stress through the passage of electrons and built-up reactive oxygen species (ROS) [20, 21] resulting in oxidative damage to the cell cycle, depletion of NAD (P)H, enzymatic inhibition, disruption of the normal electron transport chain, and specific DNA damage [22, 23]. Furthermore, pyocyanin has an antimicrobial action against bacteria and fungi as it has demonstrated an antibacterial action against several Gram-positive species, including *Staphylococcus aureus, Staphylococcus epidermis, Bacillus subtilis*, and *Micrococcus luteus*, in addition to Gram-negative organisms such as *Escherichia coli* and *Acinetobacter* [24–26]. Upon testing pyocyanin on a variety of cell lines, it has indicated an anticancer activity against various types of cancer including hepatocellular carcinoma (HepG2) [27], pelvic rhabdomyosarcoma (RD) [28], and pancreatic cancer (Panc1) [29]. The biological activities of pyocyanin are not only limited to being an antibacterial or anticancer agent, but also has several applications, where it can be used as an antibiofilm, antioxidant, antifungal, and anti-inflammatory agent. It has also been proven to be beneficial in the agricultural field as a fertilizer or pesticide [12, 24, 30].

The increase in bacterial resistance, the high numbers of cancer patients, and the emergence of aggressive types of cancer constantly urge scientists to explore alternative natural options to treat antibacterial resistance and identify effective and affordable anti-cancer treatments [24]. In this regard, pyocyanin has attracted a great attention in clinical and pharmaceutical research due to being a highly promising multi-functional agent with a pharmacological impact on both eukaryotic and prokaryotic cells [31]. Despite its various applications, pyocyanin is still an expensive compound in the market [32].

Accordingly, the objectives of the present study are to extract and characterize pyocyanin from clinical isolates of *P. aeruginosa* using an affordable and simple method. This study also sought to evaluate the antibacterial effects of pyocyanin against different bacterial isolates and compare its anticancer activities against the three most prevalent cancers; breast, lung, and colorectal cancer, using respective cell lines. The findings of our study could contribute to producing a sufficient yield of a powerful antibacterial and anticancer agent using a simple inexpensive method, depending on available media and chemicals. This, in turn, will encourage the use of natural products on a wider scale, play a role in reducing bacterial resistance to conventional drugs, and discover effective solutions to treat cancer, especially aggressive cancer.

## Methods

### Samples collection and identification

A total of 30 *P. aeruginosa* strains from different clinical specimens were first isolated from patients admitted to Mabaret Al-Asfara Hospital in Alexandria, Egypt. The samples were collected from swabs (14 isolates) from the ear, diabetic foot, bed sores, wounds and lesions, urine (six isolates), blood (four isolates), respiratory tract (five isolates), and bone tissue (one isolate). Furthermore, the isolated strains were cultured in nutrient broth and streaked on both blood and cetrimide agar plates. Initially, presumptive strains of *P. aeruginosa* were identified by routine tests including Gram-stain, colony appearance on blood agar, positive oxidase test, and pigment production on cetrimide agar. The isolates were also identified using the automated VITEK 2 Compact system (BioMérieux, France), where *P. aeruginosa* ATCC 27,853 was used as a reference strain in the identification steps.

### Antimicrobial susceptibility testing

Based on the Clinical and Laboratory Standards Institute (CLSI) guidelines, the antimicrobial susceptibility of the obtained isolates was investigated by the disk diffusion method using Muller-Hinton agar (MHA) [33]. The antibiotics included piperacillin/tazobactam (TZP, 110), ceftazidime (CAZ, 30), cefepime (FEP, 30), imipenem (IPM, 10), meropenem (MEM, 10), gentamicin (CN, 10), tobramycin (TOB, 10), ciprofloxacin (CIP, 5), and levofloxacin (LEV, 5) (Oxoid Ltd, England). The isolates that were resistant to at least one agent in three or more antimicrobial categories were considered as multidrug-resistant (MDR) according to the European Centre for Disease Prevention and Control (ECDC), where the reference strain *P. aeruginosa* ATCC 27,853 was used as a quality control [34, 35].

### Molecular identification of *P. aeruginosa*, *phzM* and *phzS*

#### DNA extraction

The DNA of each isolate was extracted by suspending a few colonies in 200 µL of sterile deionized water. Afterwards, the mixture was boiled for 10 min at 98 °C, and then the cell extract was centrifuged at 4 °C for 5 min at 15,000 xg. The DNA template for the polymerase chain reaction (PCR) was taken from the supernatant [36].

#### Amplification of 16 S rRNA, *phzM* and *phzS* genes

PCR was used to detect 16 S rRNA, *phzM*, and p*hzS*. PCR mixtures with a final volume of 20 µL consisted of 10 µL 2X TOP simple™ Dye MIX-HOT master mix (Enzynomics INC, Republic of Korea), 1 µL (5 pmole/µL) of each forward and reverse primer (Invitrogen by Thermo Fisher Scientific, USA) (Table 1), 1 µL DNA template, and 7 µL sterile water. After the preparation of samples, amplification of the genes understudy took place according to the specified thermal conditions (Table 1). In addition, two microtubes were used, one containing 1 µL of sterile deionized water instead of the DNA template, and another including the DNA of *P. aeruginosa* ATCC 27,853 served as negative control and positive control, respectively. The separation of the amplified DNA was then conducted by gel electrophoresis with 2% agarose supplemented with 0.5 µg/mL of ethidium bromide. The gel electrophoresis was run for 30 min at 130 V, which was followed by visualization under UV transillumination. Based on the fragment sizes shown in (Table 1), the amplified genes were identified in comparison to a 100 bps DNA ladder (Enzynomics Inc. - Republic of Korea) [37–39].

**Table 1:** List of primers used in this study

Primers	Target Gene	Sequence (5’- 3’)	Product Size (bp)	Amplification Conditions	Reference
16 S-F	16 S rRNA	GGGGGATCTTCGGACCTCA	956	95 °C, 10 min/30x [95 °C, 30 s-58 °C, 60 s-72 °C, 60 s] /72°C, 5 min / 4 °C, ∞	[39]
16 S-R		TCCTTAGAGTGCCCACCCG			
phzM-F	*phzM*	AACTCCTCGCCGTAGAAC	313	95 °C, 10 min/30x [95 °C, 30 s-60 °C, 30 s-72 °C, 30 s] /72°C, 5 min / 4 °C, ∞	[37]
phzM-R		ATAATTCGAATCTTGCTGCT			
phzS-F	*phzS*	TGCGCTACATCGACCAGAG	664		
phzS-R		CGGGTACTGCAGGATCAACT			

#### Pyocyanin production and extraction

The procedure detailed by Abou Raji El Feghali and Nawas [40] was used with slight modifications to extract pyocyanin. Briefly, 0.5 McFarland suspension from a fresh culture of *P. aeruginosa* was swabbed in 3 different directions on cetrimide agar plates, incubated at 37 °C for 48 h, and then the plates were left at room temperature for 5 days. Bacteria were then removed from the surface of the plates using distilled water, after which the agar was cut aseptically into small pieces and placed in a separating funnel containing chloroform. This mixture was vigorously shaken, and the emerging blue chloroform layer was removed by a pipette and filtered (using a No. 1 filter paper, double; Whatman Chemical Separation, Inc., Clinton, NJ). This step was further repeated to extract the maximum amount of pyocyanin. Afterwards, chloroform was removed from the extract using a rotary vacuum evaporator (Buchi, Switzerland) by heating it to 62 °C at 80 rpm for 2 h [41, 42].The concentrated pyocyanin was then placed in a dark container and passed through a nitrogen gas jet to ensure it was completely dry. About 40 mg of the solid mass of extracted pyocyanin was dissolved in 5 mL of sterile distilled water, and then filtered through a 0.25 μm syringe filter to make a stock concentration of 8 mg/mL. The concentrated pyocyanin was stored at -20 °C until further use [41].

#### Quantification of pyocyanin extract

The approximate concentrations of the extracted pyocyanin were calculated from the measurement of the absorbance of the acidic solutions using 0.2 M HCl at 520 nm (A_520_), multiplied by 17.072, and expressed in µg/mL [22].

### Pyocyanin characterization

#### UV-Vis spectrophotometry

A volume of 1 mL of the prepared aqueous pyocyanin extract was dissolved in 3 mL of 0.2 M HCl, then completed to reach a volume of 5 mL with the same solvent in a flask. Moreover, a blank flask was prepared using exactly the same solvents for measurement correction and avoiding relevant background. Full UV-Vis spectra for the extracted pyocyanin versus the corresponding blank were scanned using a UV-Vis spectrophotometer T80+ (PG Instruments Ltd.- England) with a scanning range of 200–800 nm. After blank subtraction, the wavelengths maxima of the extracted spectrum were distinguished and compared against the spectrum of the standard pyocyanin [11, 22].

#### Fourier Transform Infra-Red spectroscopy (FTIR)

An accurate weight of 1 mg of pyocyanin extract was encapsulated in 200 mg of KBr (Sigma-Aldrich, USA) to prepare translucent sample disks homogenized with KBr. The IR absorption spectra were obtained using a built-in plotter. At a resolution of 4 cm^− 1^, IR spectra were gathered between 400 and 4000 cm^− 1^. The functional groups and chemical bonds of the extract were investigated using a FTIR spectrophotometer (Perkin-Elmer, USA), and thoroughly compared against the standard pyocyanin spectrum [11, 41].

#### Gas Chromatography-Mass Spectrometry (GC-MS)

The GC/MS analysis was performed using a Trace 1300 GC Ultra/Mass Spectrophotometer ISQ QD (Thermo Fisher Scientific, USA) equipped with a TG-5MS Zebron capillary column (length 30 m × 0.25 mm ID, 0.25 μm film thickness). Additionally, the carrier gas, helium, was employed at a flow rate of 1 mL/min. The injector temperature was fixed at 300 °C. The oven temperature was held at 60 °C for 1 min, then increased from 60 °C to 110 °C for 2 min (9 °C/min), 110–240 °C for 5 min (7 °C/min), and 240–260 °C (20 °C/min) for 1 min. The compounds were then identified based on a comparison between their relative retention times and mass spectra with those stored in the NIST library spectra provided by the software on the GC/MS system [41, 42].

#### Liquid Chromatography-Mass Spectrometry (LC-MS)

The LC-MS analysis was performed using the 6200 series TOF/6500 series Q-TOF B.09.00 (Agilent Technologies, USA), Electrospray IonizationESI mode, positively ionized). An accurate volume of 10 µL was analyzed by LC, equipped with a C18 (1.7, 1 × 50 mm) column. The sample was gradiently eluted, with acetonitrile, at 45 °C in the reversed phase. For characterization, a comparison was made between the reported LC-MS of standard pyocyanin and those of cultural samples [11].

### Antibacterial activity of extracted pyocyanin

#### Test microorganisms

The antibacterial activity of the aqueous extracts of pyocyanin was evaluated against different clinical isolates, that were provided and previously identified by the microbiology unit of Mabaret Al-Asfara laboratories in Alexandria. They included Gram-positive bacteria (methicillin-sensitive *Staphylococcus aureus* (MSSA), methicillin-resistant *Staphylococcus aureus* (MRSA), *Staphylococcus epidermidis, Staphylococcus saprophyticus, Staphylococcus haemolyticus, Streptococcus pyogenes, Streptococcus agalactiae*, and *Bacillus sp.)*, Gram-negative bacteria (extended - spectrum beta-lactamase *Escherichia coli* (*ESBL E. coli*), extended-spectrum beta-lactamase *Klebsiella pneumoniae* (ESBL *K. pneumoniae*), *Proteus mirabilis*, and *Acinetobacter baumannii.* The reference strains obtained from the American Type Culture Collection (ATCC) were used as control including, *S. aureus* ATCC 25,923, *Listeria monocytogenes* ATCC 35,152, *Enterococcus faecalis* ATCC 29,212, *E. coli* ATCC 25,922, *E. coli* ATCC 8753, *K. pneumoniae* ATCC 10,031, *Salmonella typhimurium* ATCC 14,028, and *P. aeruginosa* ATCC 27,853).

#### Minimum inhibitory concentration (MIC) of pyocyanin

MICs were identified using the broth microdilution method in 96-well polystyrene plates [42]. A bacterial suspension of an overnight culture was prepared to equal 0.5 McFarland, and then diluted 100-fold. 50 µl of ten prepared 2-fold serially diluted concentrations of pyocyanin extract (starting with a final concentration of 8000 µg/mL) were added to 50 µL of the bacterial suspension. About 50 µL of Cation Adjusted Muller-Hinton Broth (CAMHB) were added to 50 µL of the bacterial suspension into the growth control well, and 100 µL of CAMHB were also added to the negative control well for each isolate. The microtiter plates were incubated for 16–20 h at 37 °C to ensure that microdilution tests were read more easily using colorimetric techniques based on dye reagents. The Alamar blue dye (resazurin), which is a useful growth indicator, was also used for this purpose [42, 43]. Five microliters of resazurin were then applied to each well and incubated at 37 °C for 2 h. The MIC was determined to be the lowest pyocyanin extract concentration, which prevented a change in the colour of the resazurin after incubation. The reduction of the blue dye resazurin to the pink dye resorufin in any of the wells revealed bacterial growth. All of the experiments were carried out in triplicates.

#### Minimum bactericidal concentration (MBC) of pyocyanin

To measure MBC values of pyocyanin, aliquots of 10 µL from wells, that showed inhibition in MIC, were cultured on MHA plates. After 18 to 24 h of incubation at 37 °C, the colony count was performed, and the MBC value was defined as the sample concentration generating less than 10 colonies, in which the test was conducted in triplicates [44]. The antibacterial activity was determined based on the calculated MBC/MIC ratio, where bacteriostatic effects occurred if the MBC/MIC ratio was greater than 4, as opposed to the bactericidal ones [44, 45].

### Anticancer activity

#### Cell culture

The A549 (lung adenocarcinoma cell line), MDA-MB-231 (triple negative breast cancer cell line), and Caco-2 (colorectal adenocarcinoma epithelial cell line) were obtained from the ATCC. Moreover, the skin fibroblasts were obtained from the Center of Excellence for Regenerative Medicine and Applications, Faculty of Medicine, Alexandria University. Cells were grown in Dulbecco’s modified Eagle’s medium (DMEM) with high glucose media (4.5 g/L) for cancer cell lines or with low glucose (1.5 g/L) for fibroblasts. The media was supplied with 10% Fetal Bovine Serum (FBS) and 1% penicillin/streptomycin, and then incubated at 37 °C in a humidified incubator with 5% CO_2_. A phase-contrast inverted microscope was employed to assess the cell confluence, where cell passage was conducted when cells were confluent between 70 and 90%.

#### Cytotoxicity assay

The cytotoxic activity of the pyocyanin extract was investigated using the 3-(4, 5-dimethylthiazol-2-yl)-2, 5-diphenyltetrazolium bromide (MTT) test. A549, MDA-MB-231, and Caco-2 cells were seeded at a density of 7000 cells per well in a 96-well plate, and were given 24 h to adhere. The culture media was then replaced with fresh media containing pyocyanin extract at concentrations of (50, 100, 150, 200, 400, and 600 µg/mL). Cells treated with dimethyl sulfoxide (DMSO) were considered as negative controls. Subsequently, the media in each well was discarded after 48 h, and a new medium containing MTT (0.5 mg/mL) was added. After 4 h of incubation, the supernatant was removed, and 100 µL of DMSO were added to each well to solubilize the formazan crystals that were formed. The plates were then agitated for 15 min in the dark, and a microplate reader was used to measure the absorbance at 570 nm (Tecan, USA) [46, 47]. Calculating the percentage of cell viability in comparison to the corresponding controls and determining the half-maximal inhibitory concentrations (IC_50_) from the respective sigmoidal concentration-response curve were done using the equation below [46, 47]:

$$\text{\%} \text{C}\text{e}\text{l}\text{l}\, \text{V}\text{i}\text{a}\text{b}\text{i}\text{l}\text{i}\text{t}\text{y}= \frac{\text{A}\text{b}\text{s}\text{o}\text{r}\text{b}\text{a}\text{n}\text{c}\text{e}\, \text{o}\text{f}\, \text{s}\text{a}\text{m}\text{p}\text{l}\text{e}}{\text{A}\text{b}\text{s}\text{o}\text{r}\text{b}\text{a}\text{n}\text{c}\text{e}\, \text{o}\text{f}\, \text{c}\text{o}\text{n}\text{t}\text{r}\text{o}\text{l}} \text{x} 100$$

To determine the selectivity of pyocyanin, its cytotoxicity was determined on normal fibroblasts using the same concentrations and the same incubation as mentioned above. Then, the selectivity index was calculated using the following equation [48]:

$$\text{S}\text{e}\text{l}\text{e}\text{c}\text{t}\text{i}\text{v}\text{i}\text{t}\text{y}\, \text{i}\text{n}\text{d}\text{e}\text{x}\, \left(\text{S}\text{I}\right)= \frac{{\text{I}\text{C}}_{50} \text{o}\text{n}\, \text{n}\text{o}\text{r}\text{m}\text{a}\text{l}\, \text{c}\text{e}\text{l}\text{l}\text{s}}{{\text{I}\text{C}}_{50}\, \text{o}\text{n}\, \text{c}\text{a}\text{n}\text{c}\text{e}\text{r} \, \text{c}\text{e}\text{l}\text{l}\text{s}}$$

#### Wound healing assay

The wound healing (scratch) assay was used to examine the impact of pyocyanin on the ability of A549, MDA-MB-231, and Caco-2 cells to migrate [22]. Briefly, cells were seeded at a density of 3 × 10^5^ cells per well in 6-well plates. Using a 1000 µL pipette tip, the monolayers were horizontally scraped after 24 h of incubation followed by aspiration of the culture medium. In addition, the pyocyanin extract at a concentration of 150 µg/mL was applied to A549, MDA-MB-231, and Caco-2 cells, where DMSO was used at equivalent amounts as a vehicle control. Using the inverted microscope at 100x magnification, the migratory cells were captured at various time intervals and pictures of cells and wound healing were captured at 0 and 24 h for ImageJ software analysis. The percentage of the original scratch’s width that had shrunk over time was calculated using the equation below to calculate wound closure. The experiment was carried out three times, and the findings were presented as mean ± standard error of the mean (SEM) [49].

$$\text{W}\text{o}\text{u}\text{n}\text{d}\, \text{c}\text{l}\text{o}\text{s}\text{u}\text{r}\text{e}\, \text{\%}= \frac{\text{W}\text{o}\text{u}\text{n}\text{d}\, \text{a}\text{r}\text{e}\text{a}\, \text{a}\text{t}\, 0\, \text{h}\text{o}\text{u}\text{r}-\text{W}\text{o}\text{u}\text{n}\text{d}\, \text{a}\text{r}\text{e}\text{a}\, \text{a}\text{t} 24\, \text{h}\text{o}\text{u}\text{r}\text{s} }{\text{W}\text{o}\text{u}\text{n}\text{d}\, \text{a}\text{r}\text{e}\text{a}\, \text{a}\text{t}\, 0\, \text{h}\text{o}\text{u}\text{r}} \text{x} 100$$

#### Colony forming assay

The colony forming assay was conducted as previously mentioned [50]. A549, MDA-MB-231, and Caco-2 cells were seeded in 6-well plates at a density of 500 cells/well and left to adhere for 24 h without any kind of treatment. Next, the pyocyanin extract was used to treat A549, MDA-MB-231, and Caco-2 cells at 75 µg/mL, where DMSO at an equivalent amount served as negative control. Additionally, the cells were incubated for nine days until the formation of visible colonies, and media with treatment were replaced every two days. The ImageJ software was also used to count the colonies, and their quantification was represented using GraphPad Prism version 9.

### Statistical analysis

The experiments were performed in triplicates, and the data were expressed as mean ± standard deviation (SD). The data were then analyzed using IBM SPSS software package version 20.0. (Armonk, NY: IBM Corp), employing the Chi-square test, Monte Carlo correction, and student t-test. All experiments on cell lines were analyzed by GraphPad Prism version 9 software. A t-test was used to compare the two groups, whereas the data of more than two groups were analyzed by one-way analysis of variance (ANOVA). The significance level was set at *p* ≤ 0.05.

## Results

### Samples collection, identification and their susceptibility to antimicrobial agents

30 *P. aeruginosa* isolates were collected from different clinical specimens (Table 2), and distributed according to their clinical source as well as antibiotic sensitivity. They were identified as *P. aeruginosa* by means of different phenotypic methods including: beta hemolysis on blood agar, non-lactose fermentation, positive for catalase, oxidase, and citrate tests, as well as producing pigment on cetrimide and MHA plates. Moreover, the identification was confirmed by the automated VITEK 2 compact system.

**Table 2:** Distribution of 16 S rRNA and phenazine genes (*phzM* and *phzS*) among *P.aeruginosa* isolates and concentrations of pyocyanin extracted from different samples

Isolate Code	16 S rRNA	Phenazine genes			Pyocyanin Concentration µg/mL	Specimen
		phzM	phzS	phzM and phzS		
PA1	+	+	+	+	5.036	Swab from ear
PA2	+	+	+	+	5.38	Swab from ear
PA3	+	+	-	-	-	Wound
PA4	+	+	-	-	-	Bed sores
PA5	+	-	-	-	-	Diabetic foot
PA6	+	+	+	+	4.95	Swab from Chest
PA7	+	+	+	+	7.17	Wound
PA8	+	+	+	+	5.8	Wound
PA9	+	+	+	+	5.46	Lesion
PA10	+	+	+	+	9.56	Wound
PA11	+	+	+	+	6.57	Wound
PA12	+	-	-	-	-	Lesion
PA13	+	-	+	-	-	Diabetic foot
PA14	+	-	+	-	-	Urine
PA15	+	-	-	-	-	Urine
PA16	+	-	-	-	-	Urine
PA17	+	+	-	-	-	Urine
PA18	+	+	-	-	-	Urine
PA19	+	-	-	-	-	Urine
PA20	+	+	-	-	-	Blood
PA21	+	-	+	-	-	Blood
PA22	+	-	-	-	-	Blood
PA23	+	+	-	-	-	Blood
PA24	+	+	+	+	5.3	Sputum
PA25	+	+	+	+	9.9	Sputum
PA26	+	+	+	+	5.4	Sputum
PA27	+	+	-	-	-	Bone tissue
PA28	+	+	+	+	5.9	E.T. T
PA29	+	+	+	+	5	BAL
PA30	+	+	-	-	-	Tissue Aspirate

**Fig. 2:**
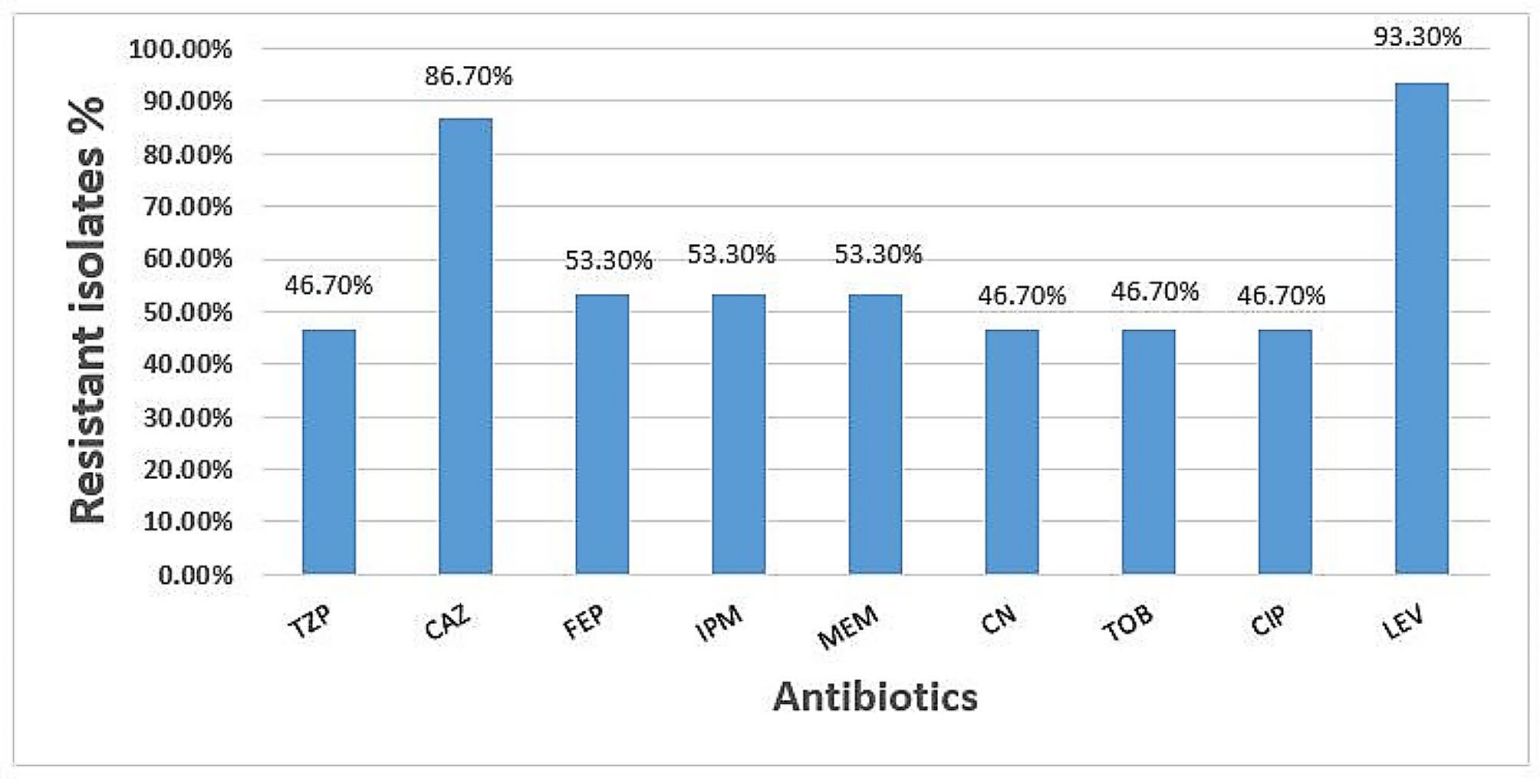
Resistance of *P. aeruginosa* isolates to the tested antibiotics

Regarding the antimicrobial susceptibility testing, 28 (93.3%) and 26 (86.7%) isolates were resistant to levofloxacin and ceftazidime, respectively. Whereas 16 isolates (53.3%) were resistant to imipenem, meropenem, and cefepime. 14 isolates (46.7%) also showed resistance to tazobactam-piperacillin, ciprofloxacin, gentamicin, and tobramycin, and about 16 isolates (53.3%) were MDR (Fig. 2).

### Molecular identification of *P. aeruginosa*, *phzM* and *phzS* genes

**Fig. 3:**
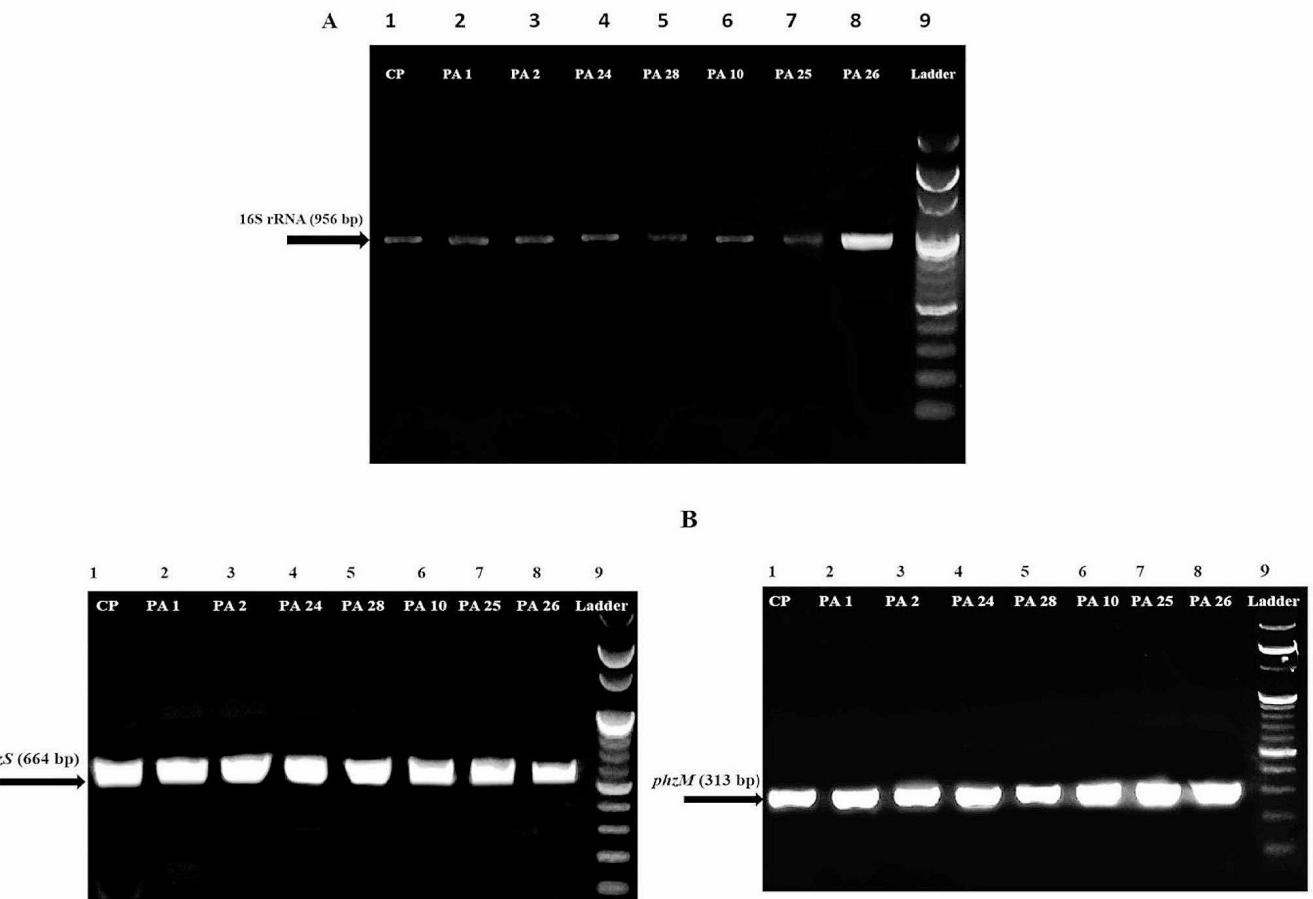
(**A**) 16 S rRNA profiles of *P. aeruginosa* isolates, (**B**) shows amplicons for phenazine modifying genes (*phzM* and *phzS*). Lane 1 (CP): positive control (*P. aeruginosa* ATCC 27,853) Lane 9 (Ladder): 100 bp ladder

The identification of the presumptive *P. aeruginosa* strains was confirmed by 16 S rRNA, and the gene was detected in all isolates (Table 2; Fig. 3A). Phenazine-modifying genes (*phzM* and *phzS*) are important genes for encoding the enzymes needed to convert phenazine-1-carboxylic acid into pyocyanin. Out of 30 isolates, 13 (43.3%) were positive for both *phzM* and *phzS* genes. It was noticed from the samples understudy that the production of pyocyanin was accompanied by the presence of both genes. Eight isolates (26.7%) were positive only for *phzM*, while three isolates (10%) had *phzS* alone. It was also observed that 6 isolates (20%) had neither *phzM* nor *phzS* genes (Table 2; Fig. 3B).

### Quantification of pyocyanin extract

About 13 isolates (43.3%) were pyocyanin producers, with a concentration ranging from 4.95 to 9.9 µg/mL. All isolates recovered from respiratory samples produced pyocyanin, while eight samples (57%) from skin and soft tissues were pyocyanin producers. On the other hand, samples from urine, blood, and bone tissue did not produce the green pigment. Furthermore, isolates PA25 (sputum) and PA10 (wound) recorded the highest pyocyanin production with concentrations of 9.9 and 9.56 µg/mL, respectively (Table 2). A statistically significant relationship was also found between respiratory, skin, and soft tissues and pyocyanin production (*p* = 0.01), while there was no significant correlation between other sources of sample and pyocyanin production (blood and urine). There was no statistically significant correlation between the source of the sample (skin, soft tissues, and respiratory samples) and the concentration of the produced pyocyanin (*p* = 0.953). Concerning the correlation between pyocyanin production and the antimicrobial resistance of the isolates, it was observed that there was a non-significant association between MDR phenotype and pyocyanin production (*p*-value = 0.153).

### Pyocyanin characterization

#### UV-Vis spectrophotometry

**Fig. 4:**
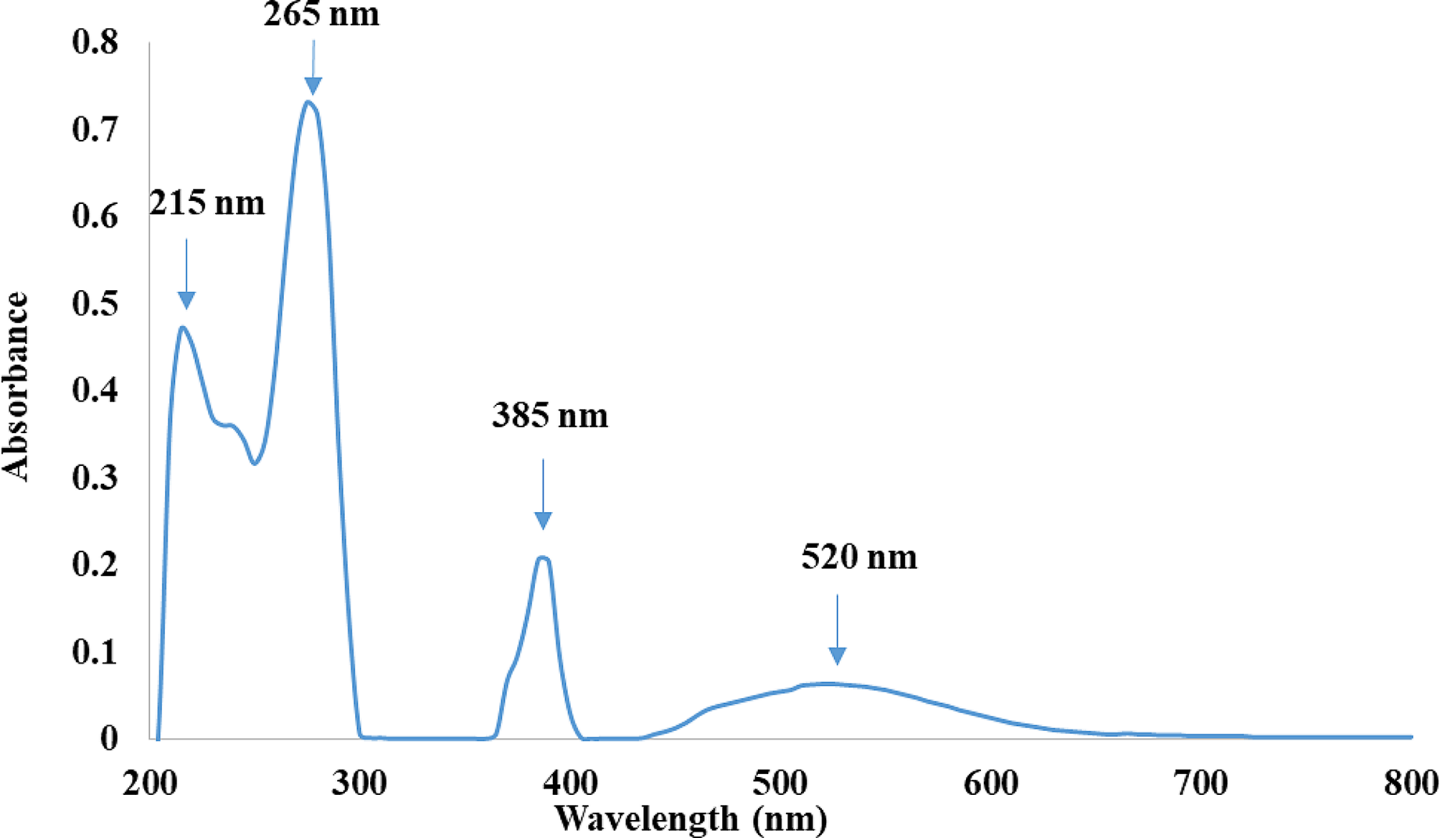
UV-Vis spectrum of pyocyanin

Pyocyanin dissolved in 0.2 M HCl exhibited absorbance maxima at 215, 265, 385, and 520 nm (Fig. 4). The pyocyanin extract, in acidic form, was quantified spectrophotometrically, depending on the measured absorbance reading at λmax of 520 nm, where concentration (µg/mL) = O.D_520_ × 17.072 [22].

#### Fourier Transform Infra-Red spectroscopy (FTIR)

**Fig. 5:**
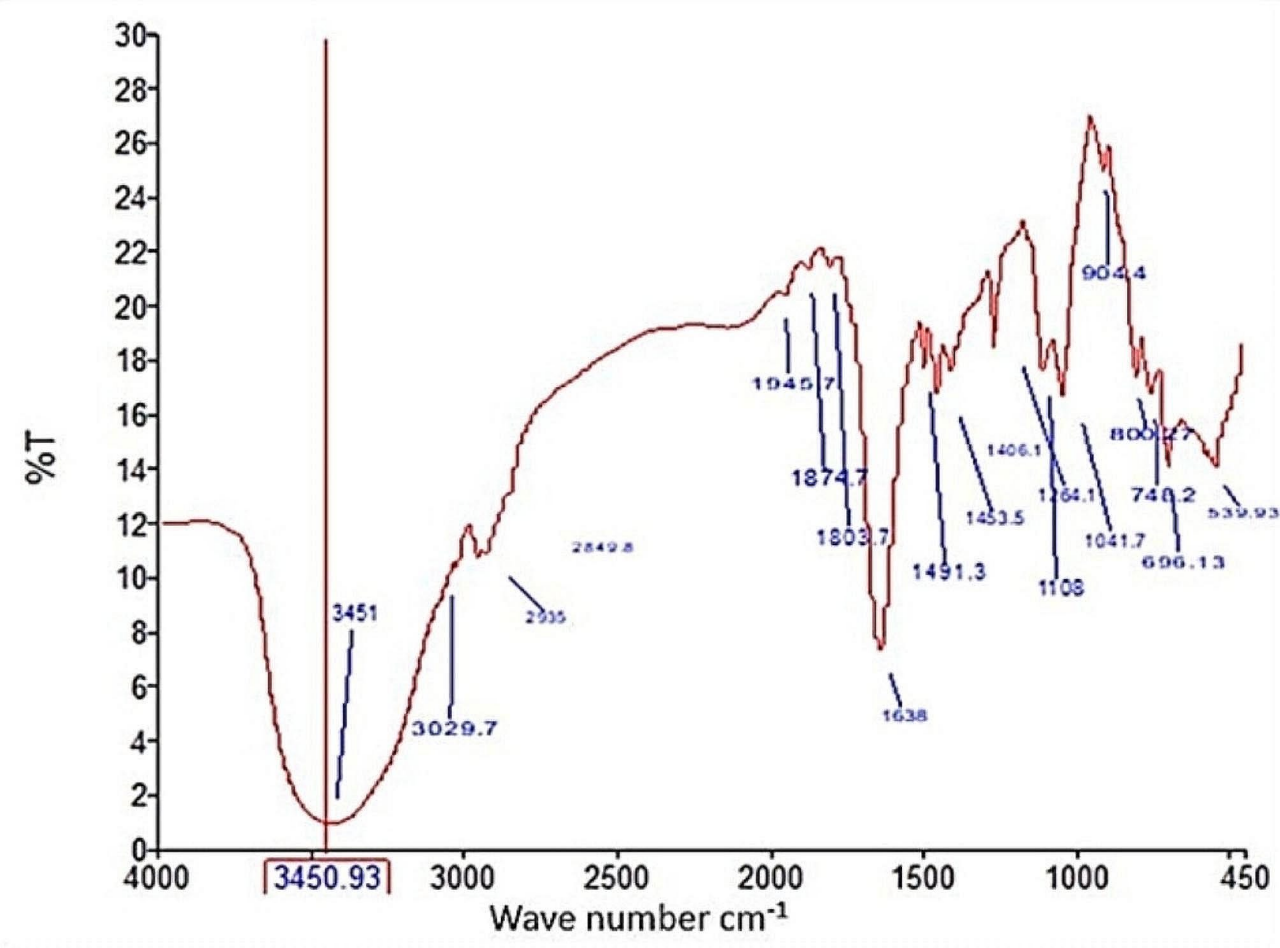
FTIR spectrum of pyocyanin produced by *P. aeruginosa*

As regards the FTIR profile analysis of the pyocyanin functional groups, phenazine was present as indicated by the molecule’s side chains. The presence of an O-H bond was shown by the peak of 3450.9 cm^− 1^, and the C-H aromatic bond was related to the peak of 2935 cm^− 1^. The C = N bond was represented by the peak of 1638 cm^− 1^, C = O-H showed a peak of 1406 cm^− 1^ and the C-N bond was represented by the peak of 1264 cm^− 1^ (Fig. 5).

#### Gas Chromatography-Mass Spectrometry (GC-MS)

**Fig. 6:**
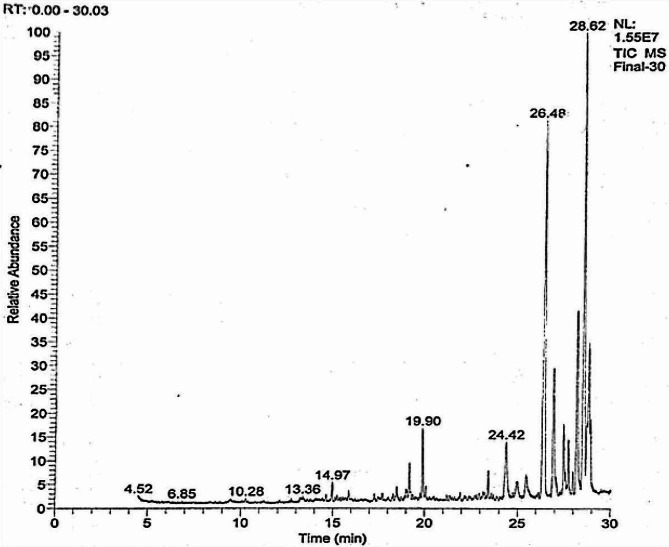
GC-MS chromatogram showing the different peaks for the pyocyanin extract

The GC-MS analysis of the methanolic pyocyanin extract showed various peaks (Fig. 6); phenol 2,4, bis (1,1, dimethyl ethyl), octane, heptadecane, dodecene, imidazole, cyclodecane, azabicyclo octane, 1-hydroxy phenazine, tetratertacontane, pentadecanoic acid, pyrrolo [1,2 A] pyrazine-1, 4-dione, hexahydro-3-(2-methyl propyl), and cyclohexane at 19.9, 20.07, 23.44, 24.41, 24.98, 25.48, 26.97, 27.49, 27.58, 27.75, 28.62, and 28.94 min, respectively.

The highest peak recorded a retention time of 28.62 min. Its chemical formula was C_11_H_18_N_2_O_2_ with a molecular weight 210. The elucidated IUPAC name was pyrrole (1, 2 A) pyrazine-1, 4-dione, hexahydro-3-(2-methyl propyl), which potentially indicated a phenazine compound.

#### Liquid Chromatography-Mass Spectrometry (LC-MS)

**Fig. 7:**
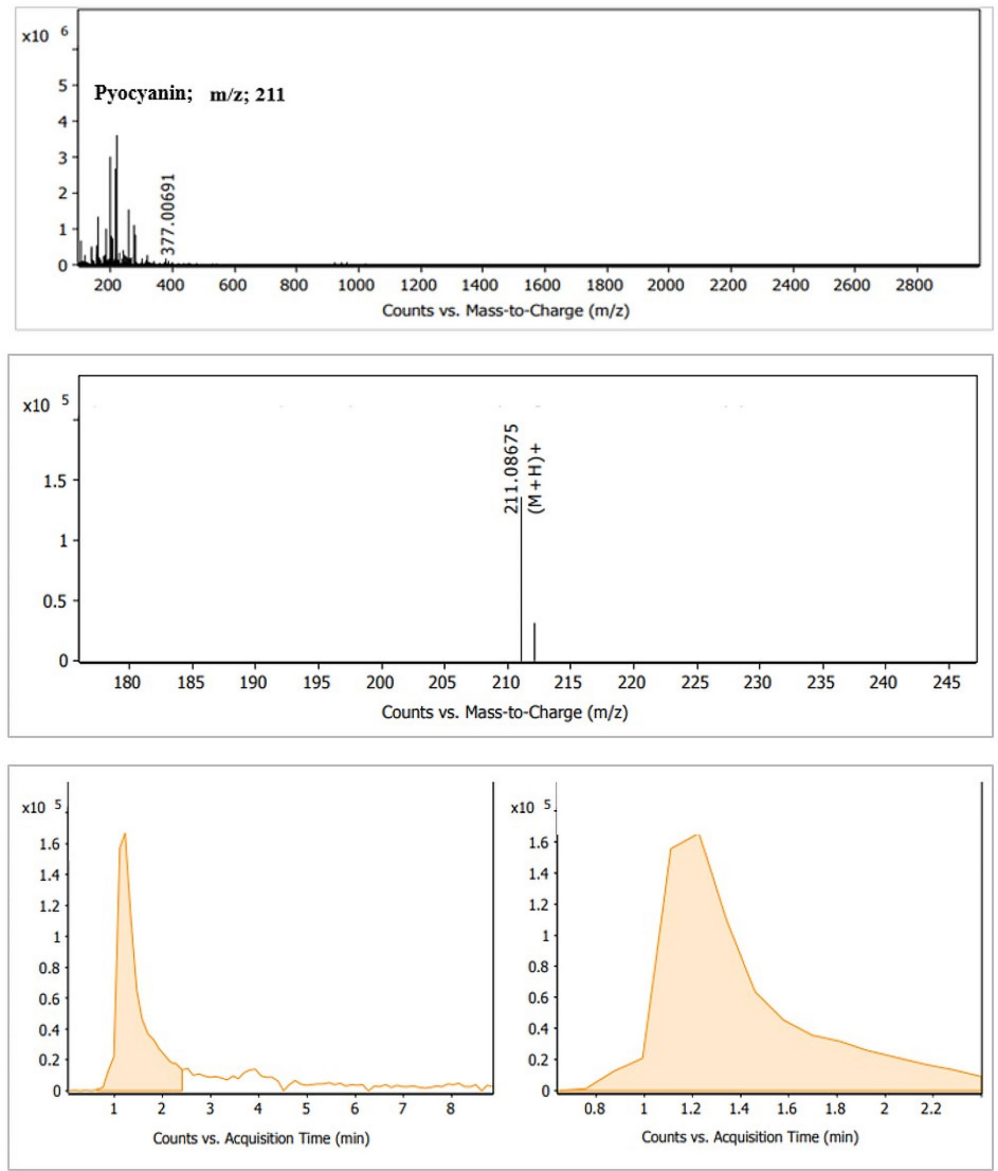
Typical LC-MS chromatogram of extracted pyocyanin

The LC-MS analysis of methanolic pyocyanin extract resulted in a series of compounds with differing sequential mass-to-charge ratios. The mass-to-charge ratio (m/z) of 211 that indicated the pyocyanin compound, was eluted at 1.20 min. (Fig. 7)

#### Antibacterial activity of pyocyanin extract

The pyocyanin extract showed an antibacterial activity against all tested bacteria at different concentrations depending on the microorganism tested, where it exhibited a more potent action against Gram-positive than Gram-negative bacteria. Moreover, the maximum activity of pyocyanin extract was recorded against *Bacillus*, *Staphylococcus*, and *Streptococcus* species, with MICs ranging from 31.25 to 125 µg/mL. Nevertheless, pyocyanin extract showed activity against *L. monocytogenes* and *E. faecalis* at higher concentrations (2000 µg/mL). *E. coli* isolates were also found to be the most sensitive Gram-negative bacteria with a MIC range of 250 to 1000 µg/mL. Pyocyanin was also active against other Gram-negative bacteria, but with high MICs (2000–4000 µg/mL). Among all tested concentrations, *P. aeruginosa* was the only tested organism to resist pyocyanin. Regarding the MBC values of pyocyanin, it was found that the extract was more lethal against Gram-positive with lower MBCs in comparison to Gram-negative bacteria, where it showed bactericidal activity against *S. aureus* ATCC 25,923, MSSA, *S. saprophyticus*, and *Bacillus* species with MBC ranging from 62.5 to 500 µg/mL. Moreover, the extract exhibited bacteriostatic activity against MRSA, other *Staphylococci* and *Streptococci* species, while its antibacterial effect was not detected against *L. monocytogenes* and *E*. faecalis. As for Gram-negative bacteria, the compound was bacteriostatic against *E. coli* species, and its effect against other Gram-negative bacteria was not detected (Table 3).

**Table 3:** Minimum bacteriostatic concentration (MIC), Minimum bactericidal concentration (MBC), MBC/MIC ratio and the antimicrobial activity of pyocyanin extract against tested Gram-positive, Gram-negative bacteria

Gram Type	Bacterial strain	MIC (µg/mL)	MBC (µg/mL)	MBC/MIC ratio	Antibacterial Effect (Bactericidal/Bacteriostatic)
**Gram Positive**	*S. aureus* ATCC 25,923	62.5	125	2	Bactericidal
	MSSA	62.5	250	4	Bactericidal
	MRSA (Swab from abdominal wall)	62.5	500	8	Bacteriostatic
	MRSA (sputum)	125	1000	8	Bacteriostatic
	MRSA (Swab from gluteal abscess)	125	1000	8	Bacteriostatic
	*S. epidermidis* (urine)	62.5	> 4000	> 4	Bacteriostatic
	*S. epidermidis* (aspirate)	62.5	> 4000	> 4	Bacteriostatic
	*S. saprophyticus*	125	250	2	Bactericidal
	*S. haemolyticus*	125	2000	16	Bacteriostatic
	*S. agalactiae*	125	4000	32	Bacteriostatic
	*S. pyogenes*	125	4000	32	Bacteriostatic
	*Bacillus* sp.1	31.25	125	4	Bactericidal
	*Bacillus* sp.2	125	500	4	Bactericidal
	*Bacillus* sp.3	62.5	62.5	1	Bactericidal
	*L. monocytogenes* ATCC 35,152	2000	> 4000	Not detected	Not detected
	*E. faecalis* ATCC 29,212	2000	> 4000	Not detected	Not detected
**Gram Negative**	*E. coli* ATCC 25,922	250	> 4000	> 4	Bacteriostatic
	*E. coli* ATCC 8753	500	> 4000	> 4	Bacteriostatic
	ESBL *E. coli* (Aspirate)	500	> 4000	> 4	Bacteriostatic
	ESBL *E. coli* (Urine)	1000	> 4000	> 4	Bacteriostatic
	*K. pneumoniae* ATCC 10,031	4000	> 4000	Not detected	Not detected
	ESBL *K. pneumonia*	4000	> 4000	Not detected	Not detected
	*A. baumannii*	4000	> 4000	Not detected	Not detected
	*P. mirabilis*	4000	> 4000	Not detected	Not detected
	*S. typhimurium* ATCC 14,028	2000	> 4000	Not detected	Not detected
	*P. aeruginosa* ATCC 27,853	No inhibition detected	Not tested	Not detected	No activity detected

### Anticancer activity

#### Cytotoxicity assay

**Fig. 8:**
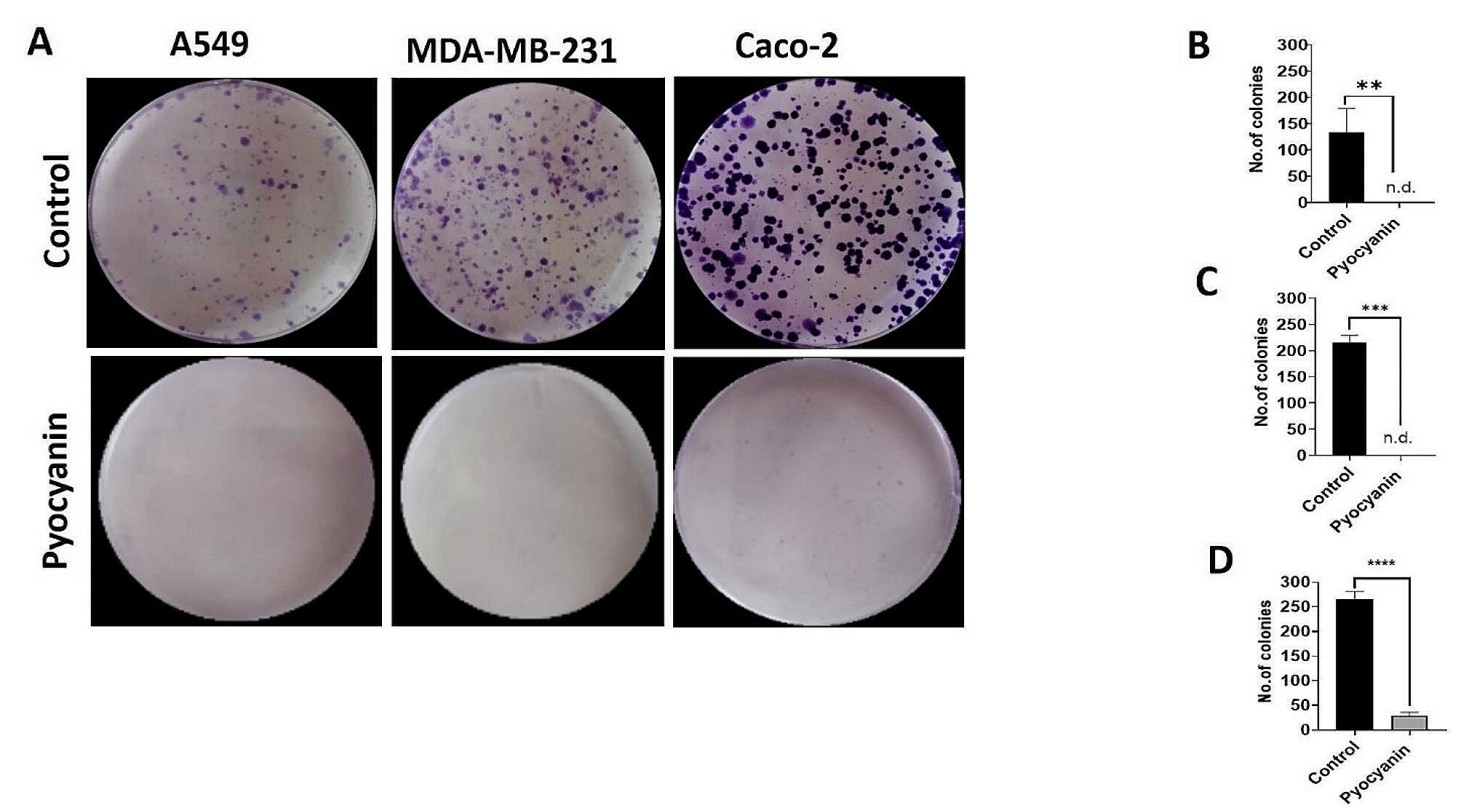
Cell viability of **(A)** A549, **(B)** MDA-MB-231, **(C)** Caco-2 cells and **(D)** normal fibroblasts after treatment with pyocyanin extract. After 24 h (24, 48 and 72 h with A549), MTT assay was performed and percentage cell viability was calculated. GraphPad Prism 9 was used to calculate the IC_50_

A549 cells were treated with various concentrations of pyocyanin ranging from 50 to 600 µg/mL for 24, 48, and 72 h. Pyocyanin was cytotoxic to the cells in a dose and time dependent manner. As shown in Fig. 8A, the 24-hour treatment did not inhibit 50% of the cells, whereas the IC_50_ after 48 h was 130 µg/mL, and after 72 h was markedly lower (IC_50_ = 45 µg/mL). To evaluate the cytotoxic effect of pyocyanin on other cancer cells, breast cancer cells MDA-MB-231 and colorectal cancer cells Caco-2 were treated by pyocyanin for 48 h, which had an IC_50_ of 105 µg/mL and 187.9 µg/mL, respectively (Fig. 8B and C). This indicated that pyocyanin has a stronger cytotoxic effect on MDA-MB-231 cells in comparison to A549 or Caco-2 cells. In addition, the cytotoxicity of pyocyanin was estimated on normal fibroblasts to determine whether it had a higher selectivity for cancer cells. The IC_50_ on fibroblasts was 194.1 µg/mL, which is higher than that for all tested cancer cell lines, in particular MDA-MB-231 and A549 (Fig. 8D). The calculated SI values for MDA-MB-231, A549, and Caco-2 cell lines were 1.84, 1.49, and 1.03, respectively.

#### Wound healing assay

**Fig. 9:**
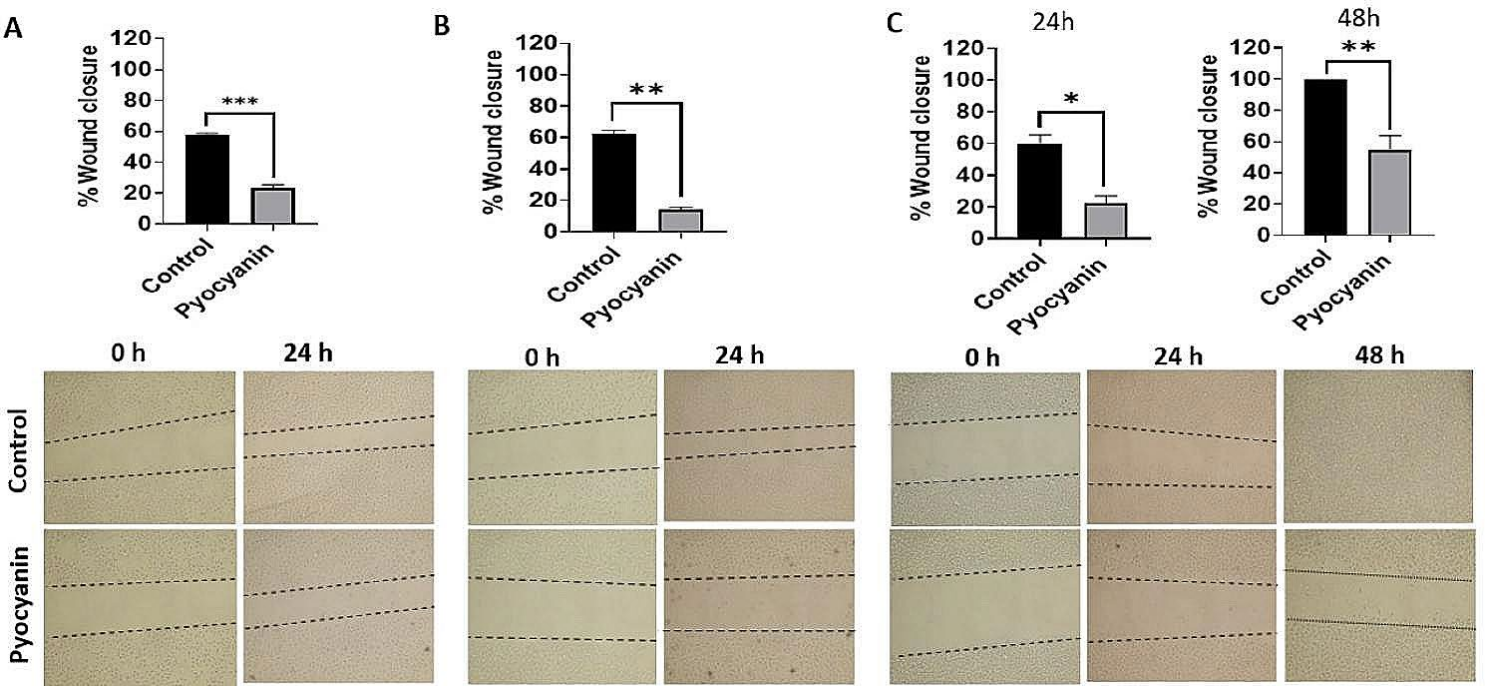
Wound healing assay on **(A)** A549, **(B)** MDA-MB-231 and **(C)** Caco-2 after treatment with 150 µg/mL of pyocyanin extract. Representative images show wound closure after treatment of A549, MDA-MB-231 for 24 h and Caco-2 for 24 and 48 h and wound closure was quantified relative to zero time. Data were expressed as mean ± SEM (*n* = 3) and statistical significance was assessed by t tests. * denotes a significant difference compared to control (*< 0.05, **< 0.01, ***<0.001 and ****<0.0001)

The impact of pyocyanin extract on the migration of the A549, MDA-MB-231, and Caco-2 cell lines was evaluated using the scratch method. The test showed that 150 µg/mL prevented the cells’ capacity to migrate, in which pyocyanin reduced the extent of inhibition in A549 cells by 35.09%. The extract also significantly reduced the migration of MDA-MB-231 cells by 47.93%. The test was conducted on Caco-2 cells for 24 and 48 h, during which pyocyanin reduced wound closure by 37.82% and 45.71%, respectively (Fig. 9).

#### Colony forming assay

**Fig. 10:**
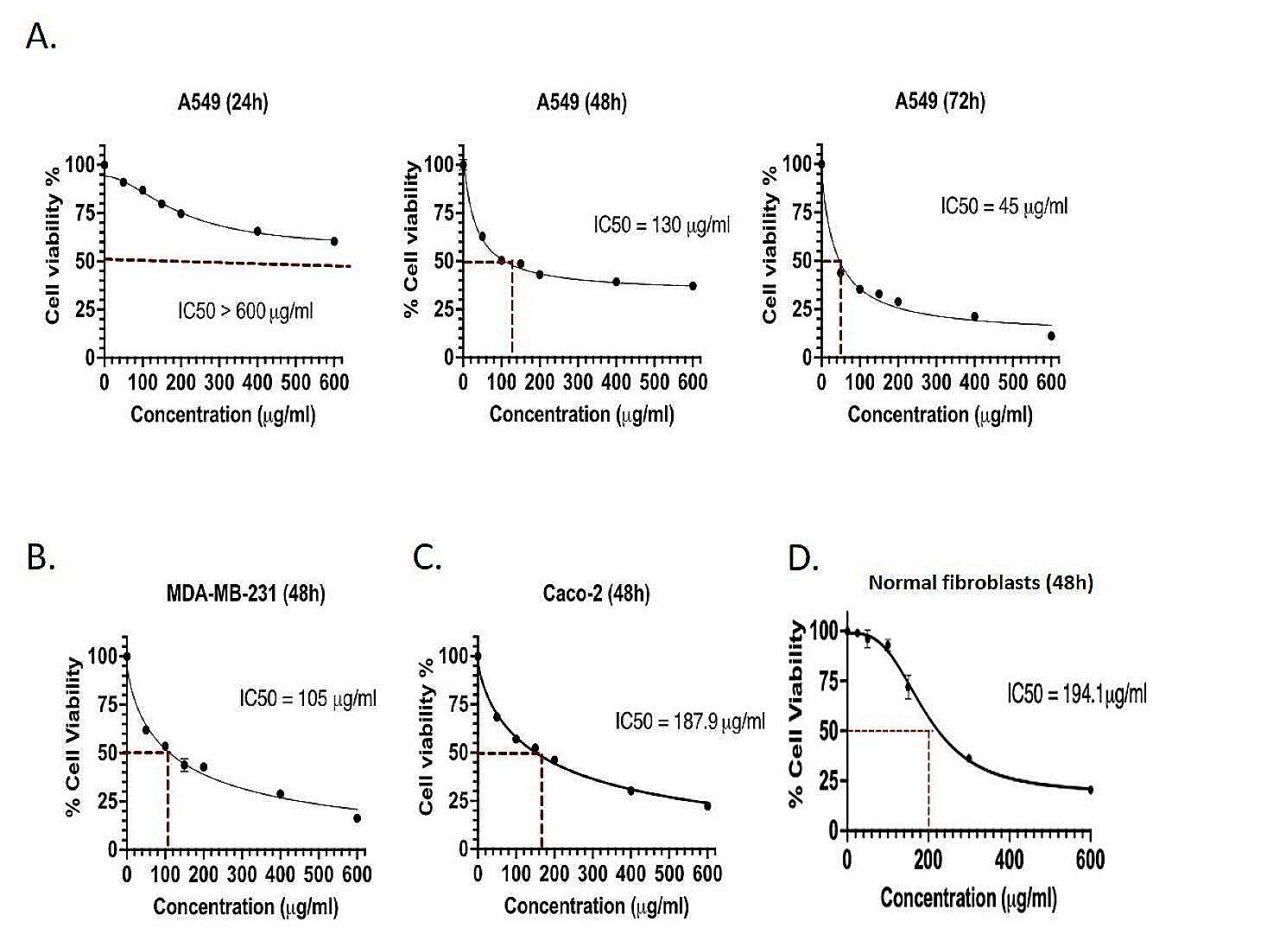
Colony forming assay on A549, MDA-MB-231 and Caco-2 cell lines after treatment with 75 µg/mL of pyocyanin extract for 9 days. **(A)** Representative images of control (upper panel) and cells treated with 75ug/mL of pyocyanin extract (lower panel). Quantification of colonies after treatment with pyocyanin in A549 **(B)**, MDA-MB-231 **(C)** and Caco-2 **(D)** represented as colony number/well. Data were expressed as mean ± SEM (*n* = 3) and statistical significance was assessed by t tests. * Denotes a significant difference compared to control (*< 0.05, **< 0.01, ***<0.001 and ****<0.0001)

The effect of pyocyanin extract on the colony formation of A549, MDA-MB-231, and Caco-2 cell lines was clarified by a colony forming assay. Each cell line was treated with 75 µg/mL of pyocyanin extract. The applied concentration highly affected Caco-2 cells, causing a noticeable drop in their colony formation ability, whereas the extract showed complete suppression of the clonogenicity in both A549 and MDA-MB-231 cell lines (Fig. 10).

## Discussion

*P. aeruginosa* is a significant nosocomial pathogen with intrinsic resistance to a wide range of antibiotics. Most infections caused by *P. aeruginosa* are due to MDR isolates [51]. It is a highly virulent bacterium armed with a variety of virulence factors [52]. In addition, it has attracted substantial interest for being one of the most economically valuable microbial species that are capable of biosynthesizing redox-active phenazine derivatives, specifically pyocyanin [31].

The antimicrobial sensitivity testing showed that more than half of the isolates (53%) had MDR. These results were somewhat consistent with those of Seiffein et al. [53]. They reported that most isolates were MDR, where all of them were levofloxacin resistant, 50% of them showed resistance to gentamicin and meropenem, and 40% were resistant to imipenem and tobramycin.

Regarding the production of pyocyanin, phenazine-1-carboxylic acid is transformed into pyocyanin by the enzymes encoded by the *phzM* and *phzS* genes. The results of the present study reported that only isolates harbouring both genes (13 isolates, 43.3%) produced pyocyanin, were in agreement with a previous study, where 33.3% of isolates had both genes, *phzM* and *phzS* were demonstrated solely in 40% and 6.6% of isolates respectively, while 20% of isolates were negative for both genes [37]. The high concentration of extracted pyocyanin, (from 4.95 to 9.9 µg/mL) in this study aligned with a previous study in Egypt, in which the pyocyanin production was from 5.9 to 9.3 µg/mL [32].

Previous studies have highlighted the critical involvement of both *phzM* and *phzS* expression in pyocyanin biosynthesis [54, 55]. The presence and functionality of both genes are essential and operate sequentially [20]. Although other *Pseudomonas* species like *P. fluorescens*, and bacteria such as *E. coli* possess the *phzS* gene, they are incapable of synthesizing pyocyanin in the absence of *phzM* gene. Instead, they directly convert PCA to 1-hydroxyphenazine. Importantly, the function of the *phzM gene* relies on the presence of the *phzS* [12]. In addition, experimental evidence has demonstrated that the disruption of either *phzM* or *phzS* leads to the suppression of pyocyanin production in *P. aeruginosa* [19, 20]. Consequently, even in cases where the *phz1* and *phz2* operons are activated, not all *Pseudomonas* species possess the ability to synthesize pyocyanin [19]. This may explain why certain isolates in the current research lacked this capability.

Quadri-technical analytical approaches including, UV-Vis spectrophotometry, FTIR, GC-MS, and LC-MS, were performed to characterize and confirm the structure of the extracted pyocyanin. The obtained UV-Vis absorbance maxima peaks of the extracted pyocyanin were in harmony with the absorbance peaks of standard pyocyanin, which showed a characteristic peak at 382 nm [11]. Moreover, the study at hand was in agreement with a previous Egyptian study, in which the standard pyocyanin showed two peaks at 262.9 and 367.1 nm, and the same peaks were also detected in pyocyanin extracted from *P. aeruginosa* clinical isolates [22]. Similarly, Ohfuji et al. [56] reported specific peaks of pyocyanin at 204, 242, 277, 387.5, and 521.5 nm when dissolved in 0.2 M HCl. Moreover, Narenkumar et al. [57] reported the pyocyanin’s characteristic peak at 278 nm. Furthermore, the UV-Vis spectrum of the extract was, to some extent, similar to the previously reported spectrum of pyocyanin dissolved in 0.2 M HCl [19].

The FTIR data demonstrated the characteristic functional groups of pyocyanin, which are responsible for its ability to bind actively to different biomolecules to perform its activity. Most of the aforementioned functional groups were previously identified in a study that demonstrated the standard pyocyanin FTIR spectrum [11]. According to Laxmi and Bhat, the FTIR analysis of pyocyanin showed the presence of O-H, aromatic C-H, C = N and C-O bonds [58].

Both the GC-MS and LC-MS analyses indicated the IUPAC name of the extracted compound to be pyrrole (1, 2 A) pyrazine-1, 4-dione, hexahydro-3-(2-methyl propyl). The compound’s molecular weight was 210 with a chemical formula of C_11_H_18_N_2_O_2_, which potentially indicated a phenazine compound. In accordance with the findings of the current study, several studies have reported on a pyocyanin from *P. aeruginosa* with a molecular mass of 210 m/z [11, 32, 59, 60]. Consequently, it is clear that there was no significant difference between the results of the four analytical techniques applied for pigment authentication.

Pyocyanin is one of the bacterial pigments that has been noted to exhibit antibacterial action against a variety of pathogens [12]. In this study, pyocyanin showed broad-spectrum activity against all tested Gram-positive and Gram-negative bacteria with different MIC values. Regarding its activity against Gram-positive bacteria, the results of the present study were aligned with previous recent studies. Shouman et al. [31] reported the remarkable efficacy of pyocyanin against MRSA, MDR *S. aureus*, *S. pyogens*, and *S. agalactiae*, with MIC values ranging from 40 to 150 µg/mL. According to Hamad et al. [41], pyocyanin was inhibitory against *B. cereus* and *S. aureus*, with MIC values of 33.3 and 58.3 µg/mL, respectively. El-Fouly et al. [32] also reported that pyocyanin with a concentration of 20 µg/mL showed maximum activity against *S. aureus*. Hence, the current study is thought to have rendered better results than other previous studies, such as that of Gahlout et al. [60], which tested the antimicrobial activity of different concentrations of pyocyanin, and reported significant activity against *Staphylococcus* isolates with MIC of 400 µg/mL. Additionally, Aziz et al. [61] investigated the sensitivity of 16 pathogenic isolates to three concentrations of pyocyanin (1125, 562, and 281 µg/mL), in which *S. aureus* isolates were sensitive to all concentrations. Interestingly, Kamer et al. [30] demonstrated the significant activity of lower pyocyanin concentrations (MIC = 8 µg/mL) against 30 MRSA clinical isolates. Furthermore, a previous study in Turkey demonstrated that pyocyanin showed maximum inhibition against *Bacillus* species in comparison to other tested organisms [23].

Regarding Gram- negative bacteria, *E. coli* isolates were found to be the most susceptible organism, followed by *S. typhimurium*. However, *K. pneumoniae*, *A. baumannii*, and *P. mirabilis* recorded the highest MIC values. In line with the findings of this study, Gahlout et al. [60] reported that *E. coli* was inhibited by 800 µg/mL, while *K. pneumoniae* was the least sensitive organism (MIC = 1000 µg/mL). The results of the study conducted by Abdul-Hussein and Atia [62] also demonstrated that pyocyanin in the concentration range from 100 to 10,000 µg/mL was most effective against *E. coli*. In accordance with Aziz et al. [61], the tested pyocyanin concentrations were effective in inhibiting the growth of *E. qcoli* and *K. pneumoniae* isolates. According to El-Shouny et al. [24], *E. coli* was sensitive, *P. mirabilis* was intermediate, while *K. pneumoniae* was resistant to pyocyanin extracted from clinical isolates. In contrast to our results, Shouman et al. [31] found that *A. baumannii* was the most sensitive Gram-negative organism, with a MIC range from 70 to 100 µg/mL, however, higher MICs of 150–300 µg/mL were found for *K. pneumoniae*, *P. mirabilis*, and *E. coli*. Generally, the variation in the reported MIC values of pyocyanin may be attributed to the difference in the nature of the tested bacterial isolates and their antibiotic sensitivity patterns [19].

In the current study, the level of MIC ranges in Gram-negative bacteria was found to be higher than that of the Gram-positive bacteria. This difference may be due to its outer membrane structure, which is exclusive to Gram-negative bacteria and is also known to contribute to antibiotic resistance. Thus, it may be necessary to emphasize that pyocyanin resistance is strongly connected to the presence of this membrane with high lipid content in the Gram-negative cell wall [63, 64].

In this study, the pigment exhibited bactericidal activity against *Bacillus*, *S. aureus* ATCC 25,923, MSSA, and *S. saprophyticus* species. A former study in China reported the possible bactericidal activity of pyocyanin against common bacteria in burn wounds including *S. aureus*, *S. epidermidis*, and *E. coli* [65], in which Pyocyanin was found to have a bactericidal effect according to the calculated MBC/MIC ratio.

This research evaluated pyocyanin’s cytotoxic effects on A549, MDA-MB-231, and Caco-2 cells. It was found that pyocyanin treatment induced a significant concentration-dependent cytotoxicity in all the studied cell lines. Additionally, it was shown to be more selective for these cancer cells than for normal fibroblasts. Pyocyanin has also shown optimum results against MDA-MB-231, which is a challenging triple-negative breast cancer (TNBC). In fact, TNBC is a difficult and aggressive complex disease that is distinguished by distinct metastatic patterns and deficiency of targeted therapies. Around 170,000 cases globally are thought to be diagnosed as TNBC, which makes up 10–20% of invasive breast cancer cases [66, 67]. According to the authors’ best knowledge, this is the first study to explore pyocyanin’s activity against MDA-MB-231 cells. A study conducted by Abdelaziz et al. [22] demonstrated that pyocyanin has significantly reduced the human breast cancer (MCF-7) cell survival in a dose-dependent manner, with an IC_50_ of 15 µg/mL against MCF-7 cells, which is less aggressive than MDA-MB-231 cells. Interestingly, Kennedy et al. [68] found that MDA-MB-231 cells were suppressed by phenazine-1-carboxamide, which is another example of phenazine derivatives; the results showed that the IC_50_ values were 62.97 ± 1.87µM. Moreover, the current results of migration and colony formation were most effective on MDA-MB-231 cells. Based on the above findings, pyocyanin can be a promising therapeutic option for TNBC.

Lung cancer is the main cause of cancer mortality and the second most prevalent type of cancer, with 1.8 million deaths and 2.2 million new cases [4]. The pyocyanin extract has also shown considerable results against A549 cells. In agreement with the results at hand, Kohatsu et al. [69] reported that pyocyanin and chloropyocyanin showed higher selectivity for A549 cells than MRC-5 normal human lung fibroblasts. In light of their remarkable selectivity, both seem to be promising candidates for the development of chemotherapeutic agents. According to O’Malley et al. [70], the cytotoxicity of pyocyanin against A549 cells was associated with cellular ATP depletion and inhibition of aconitase activity, possibly as a result of mitochondrial membrane depolarization and subsequent overexpression of superoxide dismutase. According to Kennedy et al. [68], the IC_50_ of phenazine-1-carboxamide against A549 cells was 46.78 ± 1.67 µM.

Limited studies have demonstrated the direct cytotoxic activity of pyocyanin against Caco-2 cells. Hirakawa et al. [71] reported that the adsorption of phenazines, particularly pyocyanin, hindered its cytotoxicity against both A549 and Caco-2 cells.

Pyocyanin has also shown cytotoxicity against other cancer types in previous studies. Zhao et al. [27] stated that the number of HepG2 cells declined by 67% after treatment with 10 µg/mL of pyocyanin for nine days. Moreover, Hassani et al. [28] investigated the cytotoxicity of pyocyanin on RD cells from both wild and mutant strains of *P. aeruginosa*, and discovered that the mutant strain showed greater levels of cell growth inhibition than the wild type after a 72-hour- pyocyanin therapy with IC_50_ values of 225 and 57.3 µg/mL after a 48-hour treatment. Furthermore, Moayedi et al. [29] found that pyocyanin extracted from a clinical sample was more cytotoxic against Panc-1 cells than pyocyanin extracted from an environmental sample, with IC_50_ of 118.5 and 287.4 µg/mL, respectively. According to a recent study, pyocyanin extracted from clinical and environmental isolates exhibited cytotoxic activity against colorectal carcinoma, colon cancer (HCT-116), epithelioid carcinoma (Hela), prostate cancer (PC-3), and hepatocellular carcinoma (HepG2) and human breast cancer (MCF-7). Even though both extracts were effective, the pyocyanin extracted from the clinical source was more potent than that from the environmental isolate [31]. In a prior experimental study, Patil et al. [72] found that pyocyanin’s concentrations required to inhibit cell proliferation varied when applied to different cell lines, indicating that the anticancer impact of pyocyanin is type-specific. This variation has been linked to differences in physiology, metabolism, and genetic makeup between cell lines [19, 72].

In this study, pyocyanin inhibited colony formation completely even at half IC_50_ (75 µg/mL) in A 549 and MDA-MB-231 cells. The migratory capacity was hampered by pyocyanin in all three tested cell lines, suggesting an inhibitory role of pyocyanin on metastasis.

The mechanism of antibacterial action of pyocyanin involves multiple targets and pathways. Being a redox-active molecule, pyocyanin generates reactive oxygen species (ROS) such as hydrogen peroxide (H_2_O_2_) and superoxide anion (O_2_^−^) [12, 19]. Such highly reactive molecules can cause direct oxidative damage to vital bacterial cell molecules, including nucleic acids, proteins, and lipids [19, 73, 74]. Damage to the nucleic acid may result from breaks of the nucleic acid strand, base modifications, or crosslinking between strands, leading to the instability of the bacterial genome, and inhibition of DNA replication, transcription as well as repair processes [19]. Moreover, the generated oxidative stress on various proteins can interfere with several cellular processes. The lipid peroxidation that might be caused by the generated ROS can also disrupt the bacterial cell membranes, destabilizing their integrity, and selective permeability, thus leading to leakage of crucial intracellular components [73]. In addition, pyocyanin disrupts the bacterial electron transport chain (ETC) by accepting electrons from various electron donors, such as NADH, disrupting the normal flow of electrons, leading to impairment of ATP synthesis and consequently a decrease in bacterial cell energy production. Furthermore, pyocyanin has been reported to interfere with bacterial quorum sensing signaling systems, attenuating bacterial virulence such as biofilm formation, rendering the bacteria more susceptible to clearance [19, 30]. All these factors can impair bacterial growth and survival, leading to ultimate bacterial cell death.

Similar to the antibacterial effect of pyocyanin, previous studies have attributed its anti-cancer effect mainly to its ability to induce oxidative stress within cancer cells [19, 27, 29]. Augmented oxidative stress, mainly mediated by reactive oxygen species (ROS), increased the activity of superoxide dismutase and catalase [27], which drove subsequent cell death mechanisms. The pyocyanin-induced cell damage increased cytotoxicity by activating necrosis pathways as well as caspase-dependent apoptosis pathways, as observed in a study on pancreatic cancer cells [29]. It is worth mentioning that basal levels of ROS tend to be higher in cancer cells than normal cells, which might explain the selective effect of pyocyanin on cancer cells [75].

The results provide a workflow for the production of a sufficient yield of a potent antibacterial and anticancer agent utilizing a straightforward, low-cost method depending on accessible media and chemicals. This will therefore promote the use of natural microbial products more widely, aid in the decrease of bacterial resistance to conventional antibiotics, and contribute to the development of efficient cancer treatment methods, particularly for aggressive cancer types. Furthermore, it is known that traditional therapies for cancer and infections, including chemotherapy, radiation, and antibiotics may harm healthy cells and result in systemic adverse effects and medication resistance. Thus, pyocyanin could be a good alternative for the treatment of bacterial infections and the management of both primary and metastatic cancers.

The current study faced some limitations related to testing the antibacterial activity of pyocyanin on a wide range of clinical samples with different resistance patterns, evaluating its anti-quorum sensing and antibiofilm as well as its antifungal effects. Furthermore, the cytotoxicity and antioxidant activity against more cell lines could not be assessed in the present study.

Future research on the use of pyocyanin as an antibacterial and anticancer agent can show significant promise. Nonetheless, comprehensive and extensive investigations are still needed to elucidate the precise molecular mechanisms underlying both pyocyanin’s antibacterial and anticancer activities. Exploring and understanding such mechanisms can aid in developing novel, potent pyocyanin-based therapies and synergistic combination treatments, either with traditional antibacterial or chemotherapeutic anti-cancer agents. Through the proper assessment and the study of the safety profile, pharmacokinetics, and pharmacodynamics properties of pyocyanin in vitro and in vivo, the development of proper delivery systems would be facilitated in order to enhance its efficacy and minimize any potential side effects. Conclusively, continuous research efforts are necessitated to fully employ the therapeutic potentials of pyocyanin in order to pave the way for the innovation of effective therapeutic strategies against bacterial infections and cancer.

## Conclusions

This study provided a timely and cost-effective method for the extraction of pyocyanin from clinical isolates of *P. aeruginosa*. It revealed that isolated pyocyanin was an effective broad-spectrum antimicrobial agent, as it showed inhibitory effects against the tested Gram-positive and Gram-negative bacteria, with higher activity against activity most Gram-positive species. Pyocyanin extract also showed high cytotoxicity against MDA-MB-231, A549, and Caco-2 cells. The findings of the current study indicated the efficacy of pyocyanin to inhibit colony formation and prevent the migration of the tested cell lines. Accordingly, pyocyanin may participate in the management of clinical challenges represented by the spread of bacterial infections and cancers, against which traditional treatments are powerless, being a potential therapeutic alternative option.

### Electronic supplementary material

Below is the link to the electronic supplementary material.

Supplementary Material 1

## Data Availability

No datasets were generated or analysed during the current study.

## Abbreviations

P. aeruginosa: Pseudomonas aeruginosa
AMR: Antimicrobial resistance
MDR: Multidrug resistant
MRSA: Methicillin resistant Staphylococcus aureus
ESBL: Extended spectrum beta-lactamase
CAMHB: Cation adjusted Mueller-Hinton broth
FTIR: Fourier Transform Infra-Red Spectroscopy
GC-MS: Gas chromatography mass spectrometry
MIC: Minimum inhibitory concentration
MBC: Minimum bactericidal concentration
MTT: 3-(4, 5-Dimethylthiazol-2- yl)-2,5-Diphenyltetrazolium Bromide
DMEM: Dulbecco’s modified Eagle’s medium
ROS: Reactive oxygen species
