# Supplementary material for: Transforming microbial pigment into therapeutic revelation: extraction and characterization of pyocyanin from Pseudomonas aeruginosa and its therapeutic potential as an antibacterial and anticancer agent
Source: Microb Cell Fact. 2024 Jun 13;23:174. doi: 10.1186/s12934-024-02438-6 (PMC11170807; doi:10.1186/s12934-024-02438-6)
Supplement: Supplementary file 1 — Supplementary Material 1 [file 12934_2024_2438_MOESM1_ESM.docx]

**1-Antimicrobial susceptibility testing (AST)**

**Kirby-Bauer test**


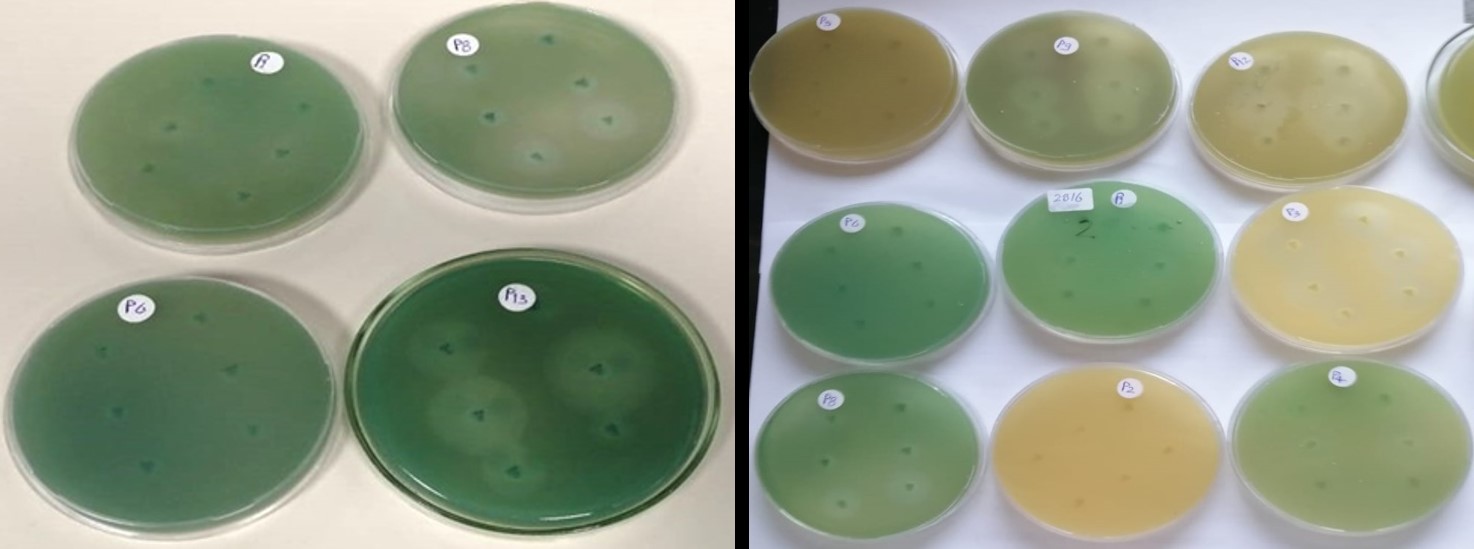


**2-Pyocyanin production and extraction**

**A-Cultivation of *Pseudomonas aeruginosa* on cetrimide Agar**


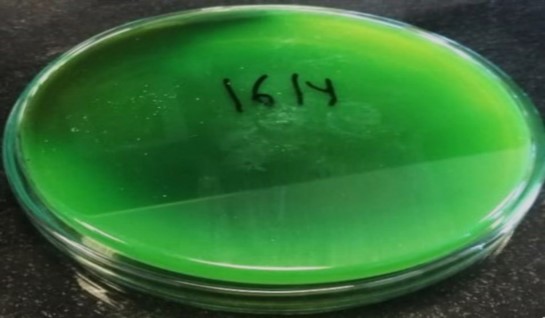


**B- Mixing pieces of cetrimide agar with chloroform in a separating funnel**

**
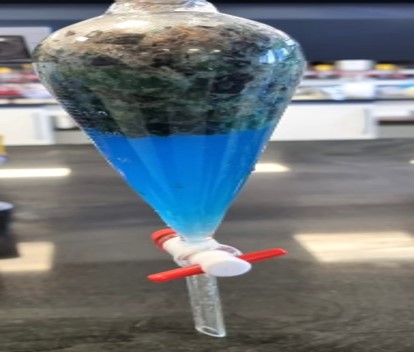
**

**C-Filtration of the blue chloroform layer**


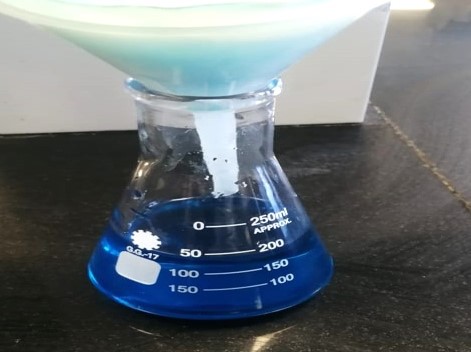


**D- Concentration of the extract in rotary vacuum evaporator**


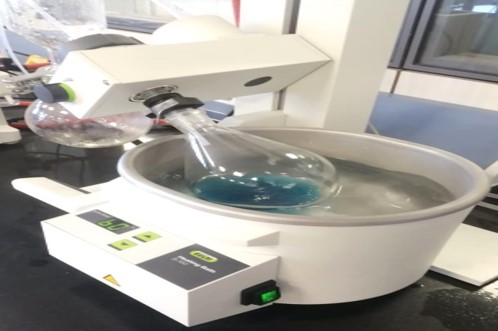


**3-Acidic solution of pyocyanin in 0.2 M HCl**


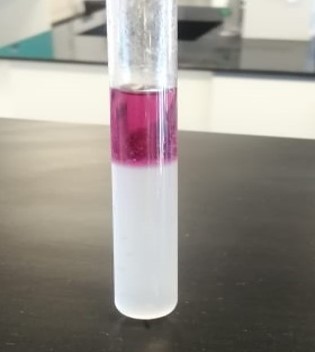


**4-UV-Visible spectrum of pyocyanin extract dissolved in 0.2 M HCl**

Spectrum **(A)** for stock pyocyanin showed characteristic peak at 520 nm that used in quantification of pyocyanin, spectrum **(B)** for diluted pyocyanin in which the characteristic UV peaks at 265 and 385 were more obvious and this spectrum used for characterization of pyocyanin.

**
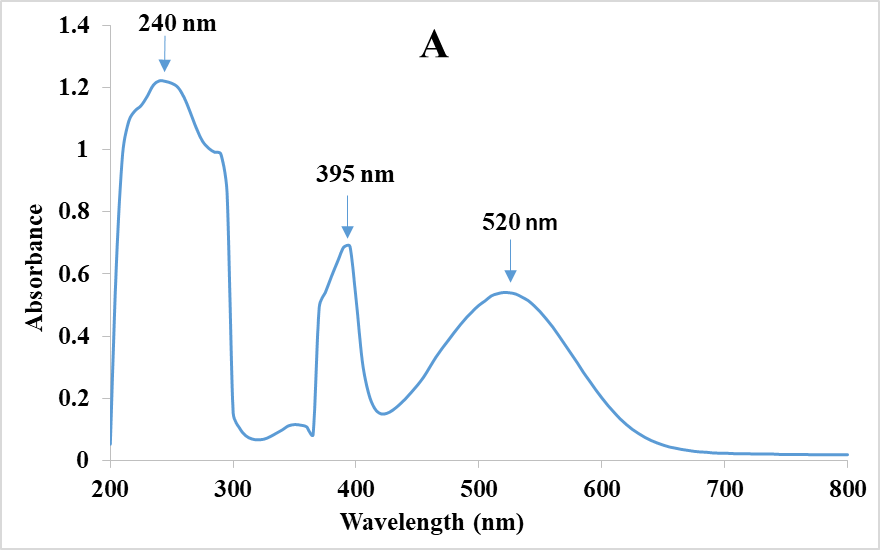
**

**
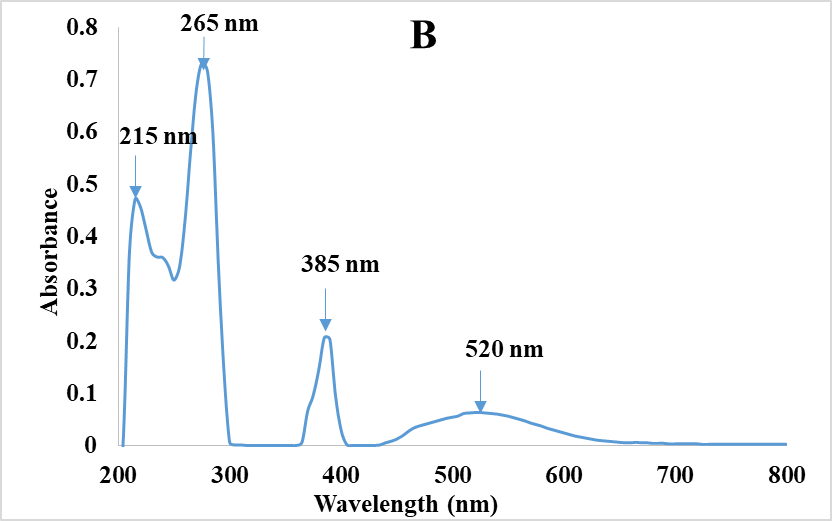
**

**5-Antibacterial activity of Pyocyanin**

**A-Antibacterial activity of pyocyanin against Gram positive bacteria ( observation of MIC by resauzurin)**

**
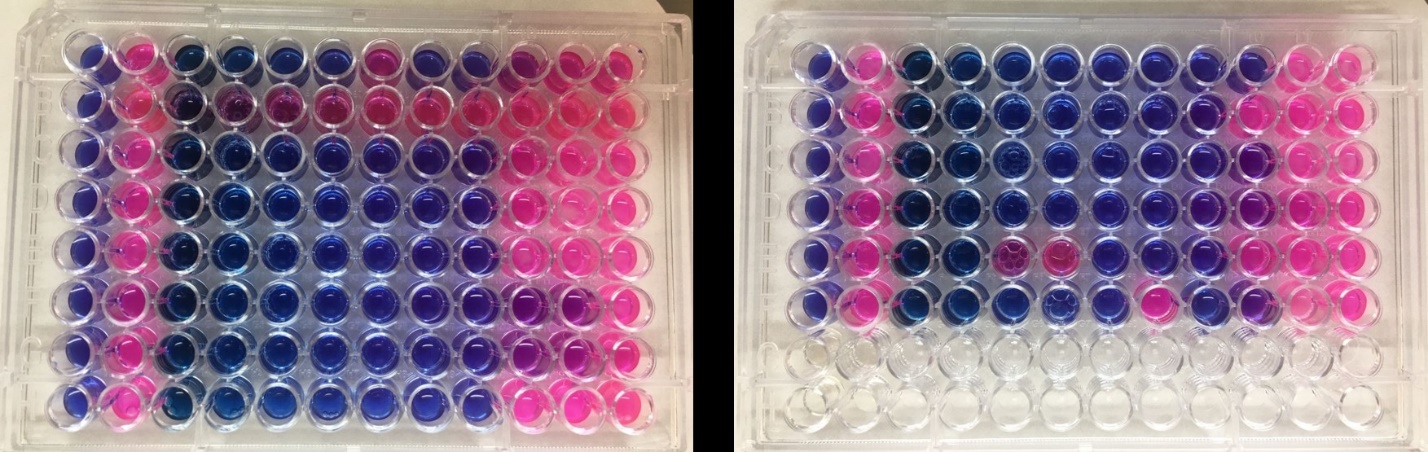
**

**B-Antibacterial activity of pyocyanin against Gram negative bacteria ( observation of MIC by resauzurin)**

**
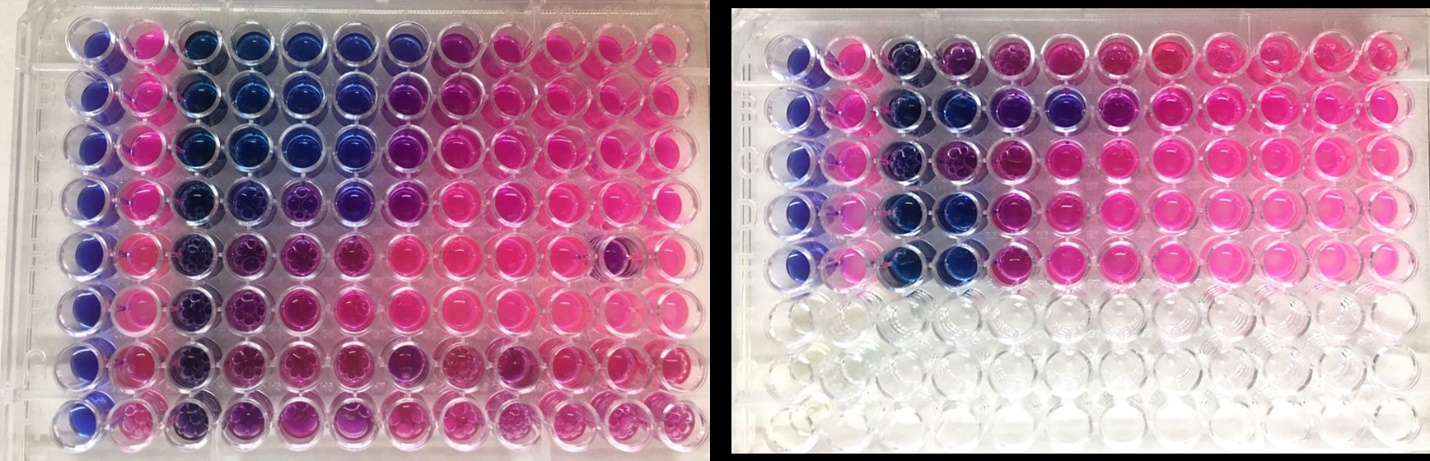
**

| **Isolate Code** | **Tazobactam - Piperacillin** | **Ceftazidime** | **Cefepime** | **Imipenem** | **Meropenem** | **Gentamicin** | **Tobramycin** | **Ciprofloxacin** | **Levofloxacin** | **Specimen** |
| --- | --- | --- | --- | --- | --- | --- | --- | --- | --- | --- |
| PA1 | 12 (R) | 16 (R) | 20 (S) | 21 (S) | 22 (S) | 15 (S) | 18 (S) | 26 (S) | 18 (R) | Swab from ear |
| PA2 | 22 (S) | 11 (R) | 21 (S) | 20 (S) | 21 (S) | 16 (S) | 17 (S) | 26 (S) | 9 (R) | Swab from ear |
| PA3 | 13 (R) | 6 (R) | 12 (R) | 6 (R) | 6 (R) | 6 (R) | 6 (R) | 6 (R) | 6 (R) | Wound |
| PA4 | 12 (R) | 6 (R) | 10 (R) | 6 (R) | 6 (R) | 6 (R) | 6 (R) | 6 (R) | 6 (R) | Bed sores |
| PA5 | 23 (S) | 15 (R) | 10 (R) | 24 (S) | 30 (S) | 17 (S) | 15 (S) | 27 (S) | 10 (R) | Diabetic foot |
| PA6 | 22 (S) | 14 (R) | 20 (S) | 25 (S) | 28 (S) | 17 (S) | 16 (S) | 26 (S) | 14 (R) | Swab from Chest |
| PA7 | 24 (S) | 13 (R) | 19 (S) | 25 (S) | 29 (S) | 18 (S) | 16 (S) | 28 (S) | 13 (R) | Wound |
| PA8 | 11 (R) | 6 (R) | 12 (R) | 6 (R) | 6 (R) | 6 (R) | 6 (R) | 6 (R) | 6 (R) | Wound |
| PA9 | 10 (R) | 6 (R) | 12 (R) | 6 (R) | 6 (R) | 6 (R) | 6 (R) | 6 (R) | 6 (R) | Lesion |
| PA10 | 23 (S) | 22 (S) | 22 (S) | 22 (S) | 26 (S) | 18 (S) | 17 (S) | 28 (S) | 12 (R) | Wound |
| PA11 | 12 (R) | 10 (R) | 10 (R) | 6 (R) | 6 (R) | 6 (R) | 6 (R) | 6 (R) | 6 (R) | Wound |
| PA12 | 25 (S) | 13 (R) | 20 (S) | 26 (S) | 27 (S) | 19 (S) | 17 (S) | 27 (S) | 12 (R) | Lesion |
| PA13 | 23 (S) | 12 (R) | 21 (S) | 24 (S) | 30 (S) | 17 (S) | 15 (S) | 28 (S) | 11 (R) | Diabetic foot |
| PA14 | 6 (R) | 6 (R) | 10 (R) | 6 (R) | 6 (R) | 6 (R) | 6 (R) | 6 (R) | 6 (R) | Urine |
| PA15 | 24 (S) | 23 (S) | 22 (S) | 13 (R) | 12 (R) | 9 (R) | 12 (R) | 17 (R) | 13 (R) | Urine |
| PA16 | 10 (R) | 11 (R) | 9 (R) | 9 (R) | 8 (R) | 6 (R) | 6 (R) | 14 (R) | 12 (R) | Urine |
| PA17 | 6 (R) | 6 (R) | 6 (R) | 10 (R) | 11 (R) | 8 (R) | 9 (R) | 13 (R) | 11 (R) | Urine |
| PA18 | 25 (S) | 24 (S) | 19 (S) | 21 (S) | 22 (S) | 17 (S) | 19 (S) | 28 (S) | 24 (S) | Urine |
| PA19 | 22 (S) | 12 (R) | 20 (S) | 22 (S) | 23 (S) | 18 (S) | 18 (S) | 13 (R) | 12 (R) | Urine |
| PA20 | 23 (S) | 22 (S) | 21 (S) | 20 (S) | 23 (S) | 17 (S) | 18 (S) | 27 (S) | 25 (S) | Blood |
| PA21 | 11 (R) | 10 (R) | 6 (R) | 6 (R) | 6 (R) | 18 (S) | 19 (S) | 27 (S) | 14 (R) | Blood |
| PA22 | 10 (R) | 10 (R) | 6 (R) | 6 (R) | 6 (R) | 11 (R) | 10 (R) | 28 (S) | 12 (R) | Blood |
| PA23 | 6 (R) | 6 (R) | 6 (R) | 8 (R) | 9 (R) | 11 (R) | 9 (R) | 27 (S) | 11 (R) | Blood |
| PA24 | 24 (S) | 11 (R) | 20 (S) | 21 (S) | 22 (S) | 18 (S) | 18 (S) | 28 (S) | 12 (R) | Sputum |
| PA25 | 25 (S) | 10 (R) | 20 (S) | 21 (S) | 20 (S) | 17 (S) | 19 (S) | 28 (S) | 12 (R) | Sputum |
| PA26 | 6 (R) | 6 (R) | 10 (R) | 11 (R) | 12 (R) | 8 (R) | 9 (R) | 14 (R) | 12 (R) | Sputum |
| PA27 | 6 (R) | 6 (R) | 6 (R) | 12 (R) | 14 (R) | 6 (R) | 6 (R) | 6 (R) | 6 (R) | Bone tissue |
| PA28 | 24 (S) | 6 (R) | 6 (R) | 10 (R) | 9 (R) | 16 (S) | 16 (S) | 10 (R) | 6 (R) | E.T. T |
| PA29 | 23 (S) | 6 (R) | 22 (S) | 20 (S) | 23 (S) | 16 (S) | 15 (S) | 28 (S) | 12 (R) | BAL |
| PA30 | 25 (S) | 6 (R) | 10 (R) | 10 (R) | 8 (R) | 6 (R) | 6 (R) | 6 (R) | 6 (R) | Tissue Aspirate |

**6-Supplementay table (Inhibition zone diameter (mm) and resistance pattern of isolates.**
